# Incidence of Schizophrenia and Other Psychoses in England, 1950–2009: A Systematic Review and Meta-Analyses

**DOI:** 10.1371/journal.pone.0031660

**Published:** 2012-03-22

**Authors:** James B. Kirkbride, Antonia Errazuriz, Tim J. Croudace, Craig Morgan, Daniel Jackson, Jane Boydell, Robin M. Murray, Peter B. Jones

**Affiliations:** 1 Department of Psychiatry, Herchel Smith Building for Brain and Mind Sciences, University of Cambridge, Cambridge, United Kingdom; 2 Department of Psychosis Studies, Institute of Psychiatry, London, United Kingdom; 3 MRC Biostatistics Unit, Institute of Public Health, University of Cambridge, Cambridge, United Kingdom; The University of Queensland, Australia

## Abstract

**Background:**

We conducted a systematic review of incidence rates in England over a sixty-year period to determine the extent to which rates varied along accepted (age, sex) and less-accepted epidemiological gradients (ethnicity, migration and place of birth and upbringing, time).

**Objectives:**

To determine variation in incidence of several psychotic disorders as above.

**Data Sources:**

Published and grey literature searches (MEDLINE, PSycINFO, EMBASE, CINAHL, ASSIA, HMIC), and identification of unpublished data through bibliographic searches and author communication.

**Study Eligibility Criteria:**

Published 1950–2009; conducted wholly or partially in England; original data on incidence of non-organic adult-onset psychosis *or* one or more factor(s) pertaining to incidence.

**Participants:**

People, 16–64 years, with first -onset psychosis, including non-affective psychoses, schizophrenia, bipolar disorder, psychotic depression and substance-induced psychosis.

**Study Appraisal and Synthesis Methods:**

Title, abstract and full-text review by two independent raters to identify suitable citations. Data were extracted to a standardized extraction form. Descriptive appraisals of variation in rates, including tables and forest plots, and where suitable, random-effects meta-analyses and meta-regressions to test specific hypotheses; rate heterogeneity was assessed by the I^2^-statistic.

**Results:**

83 citations met inclusion. Pooled incidence of all psychoses (N = 9) was 31.7 per 100,000 person-years (95%CI: 24.6–40.9), 23.2 (95%CI: 18.3–29.5) for non-affective psychoses (N = 8), 15.2 (95%CI: 11.9–19.5) for schizophrenia (N = 15) and 12.4 (95%CI: 9.0–17.1) for affective psychoses (N = 7). This masked rate heterogeneity (I^2^: 0.54–0.97), possibly explained by socio-environmental factors; our review confirmed (via meta-regression) the typical age-sex interaction in psychosis risk, including secondary peak onset in women after 45 years. Rates of most disorders were elevated in several ethnic minority groups compared with the white (British) population. For example, for schizophrenia: black Caribbean (pooled RR: 5.6; 95%CI: 3.4–9.2; N = 5), black African (pooled RR: 4.7; 95%CI: 3.3–6.8; N = 5) and South Asian groups in England (pooled RR: 2.4; 95%CI: 1.3–4.5; N = 3). We found no evidence to support an overall change in the incidence of psychotic disorder over time, though diagnostic shifts (away from schizophrenia) were reported.

**Limitations:**

Incidence studies were predominantly cross-sectional, limiting causal inference. Heterogeneity, while evidencing important variation, suggested pooled estimates require interpretation alongside our descriptive systematic results.

**Conclusions and Implications of Key Findings:**

Incidence of psychotic disorders varied markedly by age, sex, place and migration status/ethnicity. Stable incidence over time, together with a robust socio-environmental epidemiology, provides a platform for developing prediction models for health service planning.

## Introduction

Schizophrenia and other psychotic disorders exhibit variation in incidence [1], [2], prevalence [3] and course [4] along a number of dimensions, providing important signposts for clinical care, health service planning, etiological research and public health [5]. Some of these, such as variation according to genetic risk [6], family history of mental illness [7], [8] or declines in incidence with increasing age [9], are well-established and accepted in clinical and academic circles. Others, however, such as variation by place of birth and upbringing [7], migration history and minority status [10], [11], continue to court controversy [12], despite an increasingly robust empirical base [2], [11], [13]. This potentially detracts from fundamental research into the causes, prevention and treatment of psychotic disorders. Meanwhile, resolving the important issue of whether the incidence of psychotic disorders has changed over time has been hampered, despite notable efforts [14], [15], by frequent revisions to diagnostic classifications, changes in the structure of mental health service provision, evolving diagnostic fashion and imperfect control for confounders; these factors have at various time points led the scientific community to attribute importance to observations of waxing or waning rates. This trend continues to the present day [16], [17]. To advance our understanding of these issues, we had the opportunity to conduct a systematic review of the incidence of psychotic disorders in one country, England, between 1950 and 2009.

## Methods

### A. Objectives

Our principal objective was to establish a comprehensive understanding of the epidemiological landscape of psychotic disorders in England, between 1950 and 2009, by conducting a series of systematic reviews commissioned originally by the Department of Health. Four separate reviews investigated the incidence and prevalence of psychotic disorders in both population-based and non-population-based settings (i.e. institutional settings), respectively, with a fifth addressing the economic cost implications (to health services and society) associated with the prevalence of these disorders. Here, we report findings from the population-based incidence review. We specifically sought to report estimated incidence rates of psychotic disorders in England over this time period and determine whether such rates exhibited heterogeneity by

1. Age and sex
2. Ethnicity and migrant status
3. Urbanicity
4. Over time
5. Methodological quality

**Figure 1:**
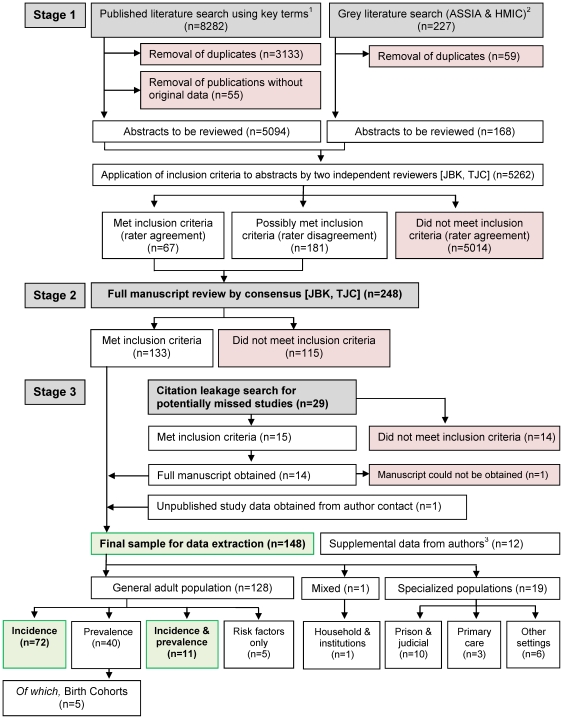
Flow diagram (selection strategy) of included studies. For the present paper, we included 83 citations which were either incidence only (n = 72) or incidence and prevalence studies (n = 11). ^1^See Methods section and ON2 for full details ^2^ASSIA: Applied Social Sciences Index & Abstracts. HMIC: Health Management Information Consortium ^3^Supplemental data was obtained in instances where the authors stated or alluded to the availability of additional relevant data, not originally published. These data were not entered as separate citations.

Our systematic reviews were designed to adhere closely to the methodological principles of the Cochrane Collaboration [18], to provide: a systematic and thorough search strategy; assessment of the methodological quality of included studies; appropriate data analysis; a clear, well-structured review, including a detailed, replicable methodology. We closely followed the guidance provided by the PRISMA statement [19], and include a copy of the PRISMA checklist (Appendix S1) and a modified version of the PRISMA flowchart (Figure 1) in our review. To this end, first we provide a detailed account of our search strategy and data extraction methodology, which allowed us to identify all citations relevant to our series of reviews. Second, we provide details of the specific statistical analyses used for our incidence-based review. To aid transparency, we have made all raw data freely available, together with the original protocol submitted to the Department of Health and other supplemental information relevant to the conduct of these reviews (www.psychiatry.cam.ac.uk/epicentre/review). We hope that this repository will act as both a source of additional information for interested readers, as well as a database to explore possible future research questions. Readers wishing to conduct possible analyses of this data will need to write a proposal for consideration by our steering committee [JBK, CM, TJC, JB, PBJ, RMM]. If accepted, this will also need to be approved by the Department of Health.

### B. Terminology

In this review, we refer to a *citation* as any unique report from the published, grey or unpublished literature. We distinguish this from a *study*, which was the identifiable project or authorship group from which a citation originated. We linked *citations* from the same study together (see ON6), but we only included data for each analysis from the citation providing the strongest data (see below).

In this review our most important figures and tables are presented with the main body of the text. Further supplemental tables and figures directly of relevance to this manuscript are published as supplemental material by the journal. Finally, additional methodological information, raw data and other ancillary data is made available through our online repository as online supplemental material (labeled ON1, ON2 etc… throughout this manuscript and available at www.psychiatry.cam.ac.uk/epicentre/review).

### C. Search strategy

A comprehensive search strategy was developed by our steering committee in consultation with an expert panel of librarians from the Evidence Adoption Centre [EAC] (part of the CLAHRC initiative), who executed the searches. The steering committee consisted of a multidisciplinary authorship team who oversaw the entire systematic review process from conception to design, analysis and dissemination. This included content area experts and methodologists with several decades of experience in psychiatric research from a range of disciplines including clinical and academic psychiatry [PBJ, JB, RMM], epidemiology [JBK, CM, PBJ, JB, RMM, TJC], geography [JBK], meta-statistics [DJ], sociology (CM] and psychology [AE, TJC].

#### C1 Inclusion criteria

Citations had to meet the following criteria to be eligible for inclusion in our reviews:

- *Time period:* Published 1950–2009
- *Extent:* Conducted wholly or partially in England
- *Scope:* Published, grey or unpublished literature
- *Contained* original data on
  ○ incident cases of non-organic adult-onset psychosis (16–64 years); or
  ○ one or more socio-environmental risk factor pertaining to incidence

We defined “original data” as data pertaining to an incidence or prevalence rate, or rate ratio between two groups. Our definition included citations with sufficient data to derive an estimate (i.e. numerator and denominator data), even if a rate had not been explicitly reported in the original citation. Derived rates were calculated and re-checked by separate members of the study team [JBK, AE].

#### C2 Literature search & citation review

To identify relevant citations, we conducted a systematic search of electronic bibliographic indexes of the published (MEDLINE, PSycINFO, EMBASE, CINAHL) and grey literature (ASSIA, HMIC) (see Table 1) to find titles or abstracts published during the period of interest, and containing a combination of a psychiatric condition term, an epidemiological term and a UK location term. These terms were developed in conjunction between the steering committee and the expert librarian group (see ON2) for full details of our search terms).

**Table 1:** Overview of bibliographic databases used to identify relevant citations.

Database		Dates covered	Scope	Website*
**1.**	**MEDLINE**	1947-	>18 m citations to journal articles in the life sciences from more than 5,400 journals	www.nlm.nih.gov/bsd/pmresources.html
**2.**	**PSycINFO**	1800-	>2.9 m citations. Systematic coverage of psychological literature, includes journals, books, and dissertation abstracts	www.apa.org/pubs/databases/psycinfo/
**3.**	**EMBASE**	1947-	>20 m citations from >7,000 biomedical journals, including >2000 not in MEDLINE	www.embase.com
**4.**	**CINAHL**	1981-	Cumulative Index to Nursing and Allied Health Literature. Indexes ∼3000 nursing and allied health journals	www.cinahl.com
**5.**	**ASSIA**	1987-	Applied Social Science Index and Abstracts. Covers health, social services, psychology, sociology and social sciences. ∼0.5 m citations from 500 journals	www.csa.com/factsheets/assia-set-c.php
**6.**	**HMIC**	1983-	Health Management Information Consortium database of clinical medicine and public health literature. >300 k citations. Combines Department of Health Library and Information Service and King's Fund Information and Library Service. Includes journals, official reports and grey literature	www.ovid.com/site/catalog/DataBase/99.jsp

&ast; Accessed 29^th^ February, 2012.

Two independent content-area experts (JBK, TJC) applied inclusion criteria to the title & abstract of all potentially relevant citations (N = 8,509, Figure 1). Each rater classified citations as having either “met initial inclusion criteria”, “possibly met inclusion criteria (further information required)” or “not met inclusion criteria”. We excluded citations which did not meet inclusion by consensus. For all remaining citations we obtained the full paper and independently re-applied the same rating criteria, with discrepant ratings (n = 41; 16.5%) resolved by the study PI [PBJ]. We identified 133 citations through this process which provided relevant incidence or prevalence data (Figure 1).

#### C3 Leakage search for missed or unpublished literature

To minimize the possibility of missing relevant data, we also appraised the bibliographies of each citation identified above, as well as reviews and meta-analyses pertinent to our objectives [11], [20], [21], [22]. We identified 15 additional citations from this process, however the full text for one citation [23] could not be obtained following an exhaustive search. Where possible, we contacted senior investigators (or other member of the study team) of any citation where there was insufficient data to determine eligibility for this review (n = 12). We clarified a citation's suitability and asked authors for any additional published or unpublished data of relevance to the review. We identified one set of unpublished data [24] in this way, (published during the course of the review [25]), yielding a total sample size of 148 citations (133+14+1), which met criteria for the review (Figure 1).

### D. Database management

All citations were collated and managed in Endnote (version 9) (file available at www.psychiatry.cam.ac.uk/epicentre/review). All manuscripts were provided by EAC librarians in paper, book or electronic format, with all necessary permissions granted prior to their distribution.

### E. Data extraction: rate-, citation- and meta-level variables

We developed a database suitable for the systematic extraction of data pertaining to rates. Its structure was guided by a previous major, international systematic review of the epidemiology of psychotic disorders [2]. Data was extracted by AE and verified for accuracy by JBK. Data extraction was managed in Microsoft Excel. We distinguished between three types of variables:

- Citation-level variables: i.e. author names, title, publication source (or unpublished), publication year, study type (incidence, prevalence, birth cohort, risk factor only or non-population-based), setting, case-finding duration, age range, diagnostic outcomes (see below), case-finding methodology, denominator source, associated citations
- Rate-level variables: Sample size (numerator), reported denominator, all incidence or prevalence rates, prevalence type (point, period, lifetime), adjustment type (crude, adjusted or standardized)
- Meta-variables: Additional indirectly derivable data from citations to permit further analyses of potential variation in rates by urbanicity, time and study quality

Rate-level data were extracted for sociodemographic variables identified during the review process, including age (bands as reported), sex, ethnicity, country of birth, geographical region and deprivation. In respect of ethnicity and country of birth, we extracted data according to categorizations from original reports. Citations referring explicitly to “country of birth” were predominantly conducted in the immediate decades following World War II, when the majority of ethnic minority groups in the UK were first generation migrants, meaning such a variable provided a proxy for ethnicity. Later studies, which needed to distinguish between first generation migrants and their British-born descendants, superseded country of birth with ethnicity as the key variable of interest. While preserving data to inspect possible differences in incidence by generation status, we also took a pragmatic decision to combine incidence data from studies of ethnicity and country of birth, when valid.

To assess possible bias in incidence reporting by study quality we constructed an index to rate the quality of each citation included in this review. The steering committee identified seven key indicators of epidemiological quality (see Box S1): a defined catchment area; accurate denominator; population-based case ascertainment; standardized research diagnoses; attempts to blind investigators to demographic variables (such as ethnicity); well-defined inclusion/exclusion criteria, and; attempts to conduct a leakage study to identify cases potentially missed by the initial screening procedure. Study quality was therefore assessed on an 8-point scale from zero to seven. Ratings were conducted by consensus between two content-area experts [JBK, AE]. Rasch modeling (TJC) suggested our index had construct validity (see ON3).

While we prioritized descriptive, systematic appraisal of data from individual studies to address the specific objectives of this review, we also had the opportunity to supplement these analyses with meta-regression to further assess whether incidence rates varied by time, urbanicity and study quality. We extracted “meta-level” data from each citation in regard to these variables. The mid-year of case ascertainment for each citation was used to appraise change in rates over time in meta-regressions. To develop an index of urbanicity we extracted a list of all settings from citations providing incidence or prevalence data in the general population, with the exception of studies conducted at the national level. This list was sent to an interdisciplinary group of investigators (JBK, TJC, PBJ, RM) who each ranked settings in terms of urbanicity. The mean of these rankings was then estimated and settings re-ranked from 1 (most urban: Hackney, Newham & Tower Hamlets – East London [10], [26]) to 38 (least urban: Chichester [27]) (see ON4).

### F. Diagnostic Outcomes

Included citations used several diagnostic classifications to estimate incidence rates of specific psychiatric disorders. Given the temporal scope of this review, there was also considerable variation in the version of each classification used (i.e. ICD-7 through to ICD-10; DSM-III, DSM-III-R, DSM-IV). We adopted a pragmatic approach to this issue by developing a diagnostic algorithm to allow us to investigate incidence variation according to broadly comparable psychotic outcomes. Thus, the lead PI (PBJ), experienced in both clinical and research-based diagnostic decision-making (PBJ) [28], [29], [30], developed an algorithm in consensus with the steering committee to classify rates in a hierarchical manner: all clinically relevant psychotic disorders, non-affective psychotic disorders, affective and substance-induced psychotic disorders, and, separately where available, schizophrenia, bipolar disorder and psychotic depression (see ON5). We chose not to analyze non-affective disorders other than schizophrenia as a separate category of disorders due to volatility of such diagnoses. In developing this algorithm we sought to maximize within-outcome homogeneity at each level of the hierarchy, while simultaneously maximizing between-outcome heterogeneity.

### G. Data analysis

#### G1 Identification of relevant data

To facilitate identification of all relevant data in this large systematic review we developed an analysis matrix to identify all citations which included suitable data for any given analysis. This allowed us to code citations on three dimensions (see schematic Figure S1): type of study (referred to as *research streams*; i.e. incidence, prevalence or data from non-population-based settings); diagnostic outcome (referred to as *research themes*) and population of interest (referred to as a *research block*: this could be an overall estimate of incidence, or rates for certain subgroups of interest, for example, by age, sex or ethnicity). >From this matrix (see ON6) many different analyses could be permuted. For each, we identified and recorded citations which contributed relevant rate data and extracted this to separate analysis files.

#### G2 Rate and citation prioritization

When a citation reported more than one type of incidence rate, crude rates (including derived rates) superseded adjusted or standardized rates, which were generally reported less often. Where two or more citations reported repeat data from the same study, the one providing the most information (including reported or derivable standard errors) was considered the *primary* citation for analyses, with other citations defined as *secondary*. Given the considerable scope of this review, it was possible for a citation to provide primary data for one analysis, but be secondary to another citation from the same study for another analysis. Where relevant, we denote primary citations in the text with an asterisk (*).

#### G3 Presentation and analyses of data

Given the scope of this review it was impractical to present results for every possible analysis. This paper focuses on those pertinent to the objectives identified above. Publication of the raw data (see ON6) permits other interested users to conduct further analyses.

Previous international systematic reviews and meta-analyses have suggested considerable heterogeneity in incidence rates along various sociodemographic and environmental dimensions [2], [11]. Indeed, using I^2^ statistics to estimate variation in rates between citations [31], we found considerable heterogeneity (typically, I^2^>90%) in our results (see below); in such circumstances meta-analyses may be inappropriate. Since such variation is also potentially relevant for etiology and health service planning, our primary objective was to preserve and report such heterogeneity, rather than pool estimates. Nevertheless, under certain circumstances the presentation of pooled estimates may be relevant to public health. We therefore took a pragmatic approach to reporting, presenting forest plots of incidence rates (and 95% confidence intervals [95%CI]) without a pooled estimate, but reporting a pooled estimate in the text for guidance, alongside the I^2^-statistic.

To facilitate meta-analytical techniques we transformed incidence estimates to their natural logarithm, appropriate for count-based data under Poisson processes. It was only possible to include incidence rates in meta-analyses where a corresponding standard error had been published or was derivable. To investigate overall incidence rates we fitted univariate random effects meta-analyses using the standard method proposed by DerSimonian and Laird [32]. A bivariate extension of this model [33] was used to investigate the effect of ethnicity on incidence rates (due to availability of data we restricted comparisons to the white, black Caribbean, black African and South Asian groups). Because there is considerable *a priori* evidence that age-specific incidence rates are modified by sex [1], [9], with a secondary peak incidence in women at approximately 45 years of age [34], we developed a fractional polynomial extension of this model to test whether there was evidence for such an interaction in the data [35]. This approach allowed us to fit non-linear associations between our outcomes of interest (psychotic disorders) and two independent predictors (age group and sex) in a meta-analytical framework to test whether there was evidence of a secondary peak incidence in women aged over 45 years old. We took the midpoint of each age stratum from citations contributing relevant data to be representative of that age group. We reported hazard ratios [HR] in men compared with women, before and after 45 years of age. These models also accounted for between-study variation using fixed study effects. We have reported the complete statistical details of this approach in a separate publication [36]. Finally, where data was sufficiently robust (>4 citations), we used meta-regression to supplement direct empirical data identified by our systematic review in respect to possible changes in incidence over time, by urbanicity and study quality. We used a suitable generalization of DerSimonian and Laird's procedure, allowing for covariate effects [37].

To inspect evidence of publication bias we conducted visual inspection of funnel plots and formal testing using Egger's test of bias in meta-analyses [38]. Given such tests are sensitive to the number of data points and between-study heterogeneity [39], we restricted such tests to meta-analyses with a minimum of ten citations.

Meta-analyses were performed in Stata (version 10) using the package *metan* and *mvmeta* for multivariate random-effects meta-analysis [40], [41], with funnel plots and Egger's bias test assessed using the *metafunnel* and *metabias* packages [42], [43]. Random effects meta-regressions were also performed in Stata using the package *metareg* [44]. Fractional polynomial meta-regressions were fitted as described by Thompson and Sharp [37], and a purpose-built R program was developed for this purpose [45]. Unless otherwise stated, all incidence rates are expressed per 100,000 person-years [100 kpy] with 95%CI where available.

## Results

**Figure 2:**
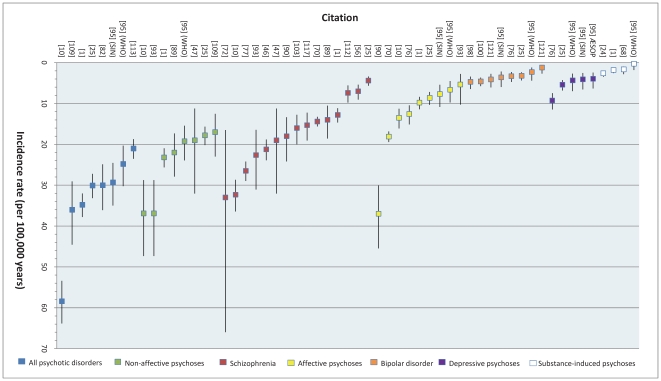
Reported overall incidence of various psychotic disorders in England, 1950–2009. The incidence of different psychotic disorders is plotted for each citation which contributed a primary rate for analysis. As the diagnostic category moves from broader (i.e. all psychotic disorders) to narrower diagnostic conditions (i.e. schizophrenia, bipolar disorder) incidence rates tend to decrease. This figure also reveals absolute differences in rates between certain conditions, for example schizophrenia vs. bipolar disorder. One identified point estimate is not shown [101] because it pertained only to rates up to age 35 years. Remaining estimates cover the full adult age range, typically until the mid-sixties.

We identified 83 citations which provided original data on the incidence of psychotic disorders in England, between 1950 and 2009 [1], [10], [14], [15], [24], [25], [26], [27], [46], [47], [48], [49], [50], [51], [52], [53], [54], [55], [56], [57], [58], [59], [60], [61], [62], [63], [64], [65], [66], [67], [68], [69], [70], [71], [72], [73], [74], [75], [76], [77], [78], [79], [80], [81], [82], [83], [84], [85], [86], [87], [88], [89], [90], [91], [92], [93], [94], [95], [96], [97], [98], [99], [100], [101], [102], [103], [104], [105], [106], [107], [108], [109], [110], [111], [112], [113], [114], [115], [116], [117], [118], [119], [120], [121]. From these, we identified 58 unique point estimates of the *overall* incidence of various psychotic disorders (Tables 1a & 1b, Figure 2). Interestingly, although some heterogeneity within outcomes was apparent, the data broadly supported our diagnostic hierarchy and is helpful in quantifying relative differences in incidence rates between disorders. Thus, incidence rates were generally highest for all syndromes, followed by non-affective psychoses, of which schizophrenia was a subset, with the incidence of affective psychoses, including bipolar disorder and psychotic depression, generally half those of their non-affective counterparts. Only four studies estimated the incidence of substance-induced psychosis; rates were generally low.

### A. Overall incidence of psychotic disorder in England

For all clinically relevant disorders, we obtained nine estimates of incidence from eight primary citations [1*], [10*], [24], [25*], [56], [68], [82*], [95*], [96], [101*], [107], [109*], [113,114*] ([95] provided estimates from two studies in Nottingham; SIN & WHO, see ON6) (Table 2 & Figure 2). Rates varied from 21/100 kpy [113] to 100/100 kpy [101]; this latter rate was estimated from an early intervention in psychosis service [EIS], which only included people up to 35 years old, and was therefore excluded from a subsequent meta-analysis. From the remaining rates, pooled incidence was estimated to be 31.7/100 kpy (95%CI: 24.6, 40.9; Figure S2). There was evidence of heterogeneity between rates (I^2^ = 0.97), unsurprising given the underlying epidemiology of psychosis [122], and for which we considered possible explanations (such as study quality, time or urbanicity; see below).

**Table 2:** Published reports of overall incidence of all psychotic disorders, non-affective psychoses and schizophrenia, England, 1950–2009.

First author	Pub. year	ID	Setting	Urban rank^2^	Mid-year (duration)^3^	Quality rank^4^	N^5^	Rate^6^	95% CI
*All psychotic disorders*									
Coid	2008	[10]	East London	1	1998 (2)	7	484	*58.4*	53.4, 63.9
Gould	2006	[82]	North London	10	2002 (1)	6	111	30.0	24.9, 36.1
Kirkbride	2006	[1]	ÆSOP^1^	21	1998 (2)	7	568	34.8	32.1, 37.8
Kirkbride	2009	[95]	Nottingham (SIN study)	25	1993 (2)	6	97	*29.3*	24.6, 35.0
Kirkbride	2009	[95]	Nottingham (WHO study)	25	1979 (2)	6	122	*24.8*	20.3, 30.3
Mahmood	2006	[101]	Lambeth (London)	8	2003 (3.2)	3	303	100.0	NA
Reay	2010	[25]	Northumberland	36	2002 (7)	4	*411*	30.1	27.2, 33.2
Rowlands	2001	[109]	North Derbyshire	31	1999 (1)	2	84	36.0	29.1, 44.6
Singh	2003	[113]	West & Southwest London	22	2000 (1)	2	295	21.0	18.7, 23.5
*Non-affective psychoses*									
Bamrah	1991	[47]	Salford	23	1984 (1)	7	14	19.0	11.3, 32.1
Coid	2008	[10]	East London	1	1998 (2)	7	362	36.8^a^	33.2, 40.8
Jablensky	1992	[89]	Nottingham (WHO study)	25	1979 (2)	6	57	22.0	17.3, 27.9
King	1994	[93]	East London	4	1992 (1)	7	62	*36.9*	28.8, 47.3
Kirkbride	2006	[1]	ÆSOP^1^	21	1998 (2)	7	378	23.2	21.0, 25.7
Kirkbride	2009	[95]	Nottingham (SIN study)	25	1993 (2)	6	80	*19.2*	15.4, 23.9
Reay	2009	[25]	Northumberland	36	2002 (7)	4	*243*	17.8	15.7, 20.2
Rowlands	2001	[109]	North Derbyshire	31	1999 (1)	2	42	17.0	12.6, 23.0
*Schizophrenia*									
Allardyce	2001	[46]	Camberwell	5	1988 (12)	7	265	*21.2*	18.8, 23.9
Bamrah	1991	[47]	Salford	23	1984 (1)	7	14	19.0	11.3, 32.1
Brewin	1997	[56]	Nottingham (SIN study)	25	1993 (2)	7	57	7.0	5.4, 9.1
Coid	2008	[10]	East London	1	1998 (2)	7	268	*32.4*	28.7, 36.5
de Alarcon	1993	[70]	Oxfordshire	35	1981 (12)	2	593	14.4	13.3, 15.6
Gater	1995	[75]	South Manchester	15	1990 (1)	3	*68*	33.0^a^	16.5, 66.0
Giggs	1973	[77]	Nottingham	25	1965 (7)	2	478	*26.5*	24.2, 29.0
Jablensky	1992	[89]	Nottingham (WHO study)	25	1979 (2)	6	48	14.0	10.6, 18.6
Jones	1991	[90]	Nottingham	25	1982 (1)	2	44	18.0	13.4, 24.2
King	1994	[93]	East London	4	1992 (1)	7	38	*22.6*	16.5, 31.1
Kirkbride	2006	[1]	ÆSOP^1^	21	1998 (2)	7	209	12.0	11.2, 14.7
McNaught	1997	[103]	Hampstead	25	1991 (∼1)	5	35	16.0	12.8, 20.0
Reay	2010	[25]	Northumberland	36	2002 (7)	4	*60*	4.4	3.4, 5.7
Shepherd	1989	[112]	Aylesbury	32	1977 (1.5)	2	49	7.4	5.6, 9.8
van Os	1996	[117]	Camberwell	5	1990 (5)	4	79	15.3	12.3, 19.1

1 ÆSOP: SE London, Nottingham, Bristol.

2 Composite perceived urbanicity rank, assessed by 4 raters (JBK, PBJ, TJC, RM). 1 = most urban, 38 = least urban.

3 Mid-year of case ascertainment period (duration in years).

4 Study quality according to criteria outlined in methodology. Min = 0, Max = 7.

5 Numbers *underlined in italics* denote a derived N – not reported in original citation but possible to derive from other provided data.

6 Crude incidence per 100,000 unless specified. *Underlined italics* denote derived rate.

a adjusted rate.

NA = Not further information provided or derivable.

The incidence of non-affective psychoses was generally lower (Table 2 & Figure 2); the pooled estimate from eight primary citations [1*], [10*], [24], [25*], [47*], [56], [65], [78], [80], [89*], [93*], [95*], [107], [109*], [ 111], [113] was 23.2/100 kpy (95%CI: 18.3, 29.5; I^2^ = 0.94; Figure S2), though this varied from 17/100 kpy [109] to 37/100 kpy [93]. We identified 15 primary estimates of the overall crude incidence of schizophrenia in England [1*], [10*], [25*], [46*], [47*], [56*], [65], [68], [69], [70*], [75*], [77*], [79], [80], [89*], [ 90*], [93*], [94], [97], [103*], [112*], [114], [117*] (Figure 2), ranging from 4.4 to 33/100 kpy (I^2^ = 0.97). As expected, the pooled incidence rate was lower than for previous outcomes (15.2/100 kpy; 95%CI: 11.9, 19.5). There was no evidence of publication bias for this outcome (Egger's p-value = 0.24; see Figure S3).

Overall, the incidence of affective psychoses was lower than for their non-affective counterparts (Table 3). We estimated the pooled crude rate [1*], [10*], [24], [25*], [70*], [76*], [78], [79], [90*], [95*], [96], [107], [ 114] to be 12.4/100 kpy (95%CI: 9.0, 17.1; Figure S2), though heterogeneity was once again substantial (I^2^ = 0.97). For bipolar disorder, where heterogeneity was moderate (I^2^ = 0.54) between the nine unique estimates from seven primary citations [25*], [49], [68], [76*], [93*], [95*], [98*], [100*], [121*], we estimated pooled crude incidence as 3.7/100 kpy (95%CI: 3.0, 4.5). For the depressive psychoses we identified less data [25*], [68], [76*], [95*], where the corresponding pooled estimate was 5.3/100 kpy (95%CI: 3.7, 7.6; I^2^ = 0.83). Finally, the pooled crude incidence of substance-induced psychoses in England was 1.9/100 kpy (95%CI: 1.2, 2.8; I^2^ = 0.63; Figure S2), identified from four data sources [1*], [24*], [68*], [95*], [114].

**Table 3:** Published reports of overall incidence of affective psychosis, including bipolar disorder and the depressive psychosis, and substance-induced psychoses, England, 1950–2009.

First author	Pub. year	ID	Setting	Urban rank^2^	Mid-year (duration)^3^	Quality rank^4^	N^5^	Rate^6^	95% CI
*Affective psychoses*									
Coid	2008	[10]	East London	1	1998 (2)	7	122	*13.5*	11.3, 16.1
de Alarcon	1993	[70]	Oxfordshire	35	1981 (12)	2	740	18.1	16.8, 19.5
Gater	1989	[76]	South Manchester	21	1977 (10)	2	114	*12.6*	10.5, 15.1
Jones	1991	[90]	Nottingham	25	1982 (1)	2	90	37.0	30.1, 45.5
Kirkbride	2009	[95]	Nottingham (SIN study)	25	1993 (2)	6	32	*7.7*	5.4, 10.9
Kirkbride	2009	[95]	Nottingham (WHO study)	25	1979 (2)	6	26	*6.7*	4.5, 9.8
Kirkbride	2006	[1]	ÆSOP^1^	21	1998 (2)	7	160	9.8	8.4, 11.4
Reay	2010	[25]	Northumberland	36	2002 (7)	4	*118*	8.6	7.2, 10.4
*Bipolar disorder*									
Gater	1989	[76]	South Manchester	21	1977 (10)	2	30	*3.3*	2.3, 4.7
King	1994	[93]	East London	4	1992 (1)	7	9	5.4	2.8, 10.3
Kirkbride	2009	[95]	Nottingham (SIN study)	25	1993 (2)	6	15	*3.6*	2.2, 6.0
Kirkbride	2009	[95]	Nottingham (WHO study)	25	1979 (2)	6	9	*2.3*	1.2, 4.4
Leff	1976	[98]	Camberwell	5	1970 (9)	3	38	4.7	3.4, 6.5
Lloyd	2005	[100]	ÆSOP^1^	21	1998 (2)	7	75	4.6	3.7, 5.8
Reay	2010	[25]	Northumberland	36	2002 (7)	4	*44*	3.2	2.4, 4.4
Wing	1976	[121]	Salford	23	1971 (5)	2	*6*	1.2	0.5, 2.7
Wing	1976	[121]	Camberwell	5	1971 (5)	2	*25*	4.1	2.8, 6.1
*Depressive psychoses*									
Gater	1989	[76]	South Manchester	21	1977 (10)	2	84	*9.3*	7.5, 11.5
Kirkbride	2009	[95]	Nottingham (ÆSOP study)	25	1998 (2)	6	17	3.9	2.5, 6.3
Kirkbride	2009	[95]	Nottingham (SIN study)	25	1993 (2)	6	17	*4.1*	2.5, 6.6
Kirkbride	2009	[95]	Nottingham (WHO study)	25	1979 (2)	6	17	*4.3*	2.7, 7.0
Reay	2010	[25]	Northumberland	36	2002 (7)	4	*74*	5.4	4.3, 6.8
*Substance-induced psychoses*									
Croudace	2000	[68]	Nottingham	25	1993 (2)	7	13	*1.6*	1.0, 2.8
Kirkbride	2009	[95]	Nottingham (WHO study)	25	1979 (2)	6	1	*0.3*	0.0, 1.8
Kirkbride	2009	[1]	ÆSOP^1^	25	1998 (2)	7	*29*	1.8	1.3, 2.6
Mitford	Unpub.	[24]	Northumberland	36	2002 (7)	4	46	*2.6*	1.9, 3.5

1 ÆSOP: Southeast London, Nottingham, Bristol.

2 Composite perceived urbanicity rank, assessed by 4 raters (JBK, PBJ, TJC, RM). 1 = most urban, 38 = least urban.

3 Mid-year of case ascertainment period (duration in years).

4 Study quality according to criteria outlined in methodology. Min = 0, Max = 7.

5 Numbers *underlined in italics* denote a derived N – not reported in original citation but possible to derive from other provided data.

6 Crude incidence per 100,000 unless specified. *Underlined italics* denote derived rate.

### B. Incidence of psychotic disorders by gender and age

For all clinically relevant psychoses [1*], [10*], [24*], [66], [107], the non-affective psychoses [1*], [10*] (supplemental data provided by authors), [24*], [56*], [65*], [74*], [107], and schizophrenia as a separate outcome [1*], [56*], [57*], [66], [81*], [118*], the available data generally indicated that incidence declined with age for both men and women, being steeper for men with a secondary peak in incidence for women, commencing in their mid- to late-forties (see, for example, Figure S4). Fractional polynomial meta-regression confirmed these interactions for non-affective psychoses and schizophrenia, independently. For schizophrenia, for example, prior to 45 years old, pooled rates were elevated amongst men compared with women (hazard ratio [HR]: 1.99; 95%CI: 1.70, 2.33), but at later ages there was no evidence for this (HR: 0.98; 95%CI: 0.70, 1.36).

A different, though analogous pattern emerged in respect of the affective psychoses, though less data was generally available [1*], [10*], [24*], [50], [57*], [76*]. Two citations reported higher rates of affective psychoses in women compared with men [57*], [76*], but a further citation [1*] reported no overall gender differences (incidence rate ratio [IRR]: 1.0; 95%CI: 0.7, 1.6). Where incidence data was available by age and gender [1*], [10*] (supplemental data provided by authors), [24*], [76*] a fractional polynomial regression suggested that prior to 45 years of age there were no significant differences in affective psychosis risk by gender (HR: 0.98; 95%CI: 0.81, 1.19), but rates were higher amongst women thereafter (HR: 1.40; 95%CI: 1.02, 1.91).

Data from studies which considered the incidence of bipolar disorder separately for men and women [76*], [98*], [100*], [108*], [115], [121*] suggested pooled rates were similar (men: 4.0/100 kpy; 95%CI: 2.9, 5.6 vs. women: 3.9/100 kpy; 95%CI: 2.1, 7.5), with little evidence of further interaction with age [48], [57*], [76], [91*], [115*]. A similar pattern was reported from published age-gender rates of depressive psychoses, identified in two citations from the same study [76], [115*].

Two citations [1*], [24*] were identified during our review process which estimated incidence of substance-induced psychoses by age and gender. In both, we obtained the original data from the authors. One study [1*] reported higher rates for men (λ = 2.5; 95% CI: 1.6, 3.8) than women (λ = 0.9; 95% CI: 0.4, 1.8), and in both samples incidence peaked in the early twenties, declining rapidly thereafter.

### C. Incidence of psychotic disorders by ethnicity

**Figure 3:**
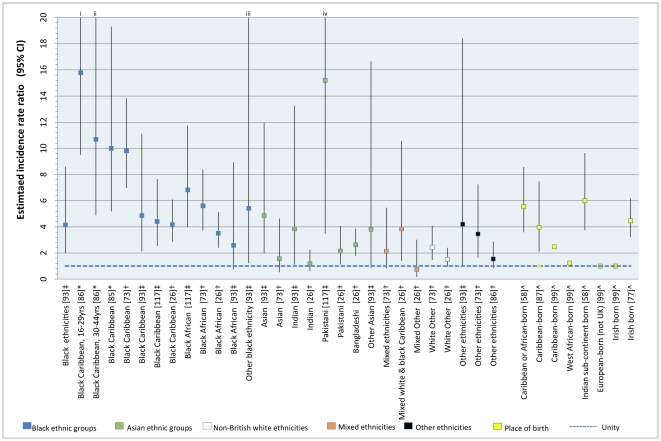
Reported incidence rate ratios of schizophrenia by ethnic group and country of birth, England, 1950–2009. Point estimates are colored by broad ethnic group. IRR are in descending order for narrow ethnic groups. Baselines: †white British; ‡white group; *Non black Caribbean; ∧UK-born. C96 did not provide data to estimate confidence intervals. i, ii, iii & iv: Upper confidence limits truncated for clarity. Actual values: i:26.2; ii: 23.4; iii: 23.6; iv: 66.5.

We identified twenty six citations [10], [14], [26], [49], [52], [58], [60], [71], [72], [73], [77], [80], [85], [86], [87], [88], [93], [97], [98], [99], [100], [102], [104], [110], [117], [120] which provided incidence data in relation to ethnicity or country of birth. Eighteen of these included data on schizophrenia [10], [14], [26*], [58*], [60], [72*], [73*], [77*], [80], [85*], [86*], [87*], [93*], [97], [99*], [102*], [104], [117*], with ten primary citations providing 37 overall incidence estimates in minority ethnic groups [26*], [73*], [85*], [86*], [93*], [117*] or by country of birth [58*], [77*], [87*], [99*] (Figure 3). Some citations also provided rates in different ethnic groups stratified by age [52*], [73*], [85*], [86*], [102*], [110*], sex [26*], [52*], [72*], [73*], [87*], [88], [102*] and generation status [10*], [86*], [102*].

Rates of psychotic disorder were most notably and consistently raised for people of black ethnicities compared with the baseline population in each study (typically those of white or white British ethnicity). For example, fifteen of sixteen relative risk estimates for schizophrenia (Figure 3) indicated significantly increased risk amongst those of black Caribbean or African origin, with point estimates ranging from 2.5 (Caribbean-born) [99] to 15.8 (black Caribbean people, aged 16–29 years) [86]. In only one study, by King *et al.* [93], was the derived relative risk in the black African group not statistically significantly greater than in the white group (RR: 2.5; 95% CI: 0.7, 8.9), but this was based on only three black African cases. We performed a random effects meta-analyses on data from five studies which presented overall incidence rates of schizophrenia in different ethnic minority groups [26*], [73*], [85*], [93*], [117*], which suggested rates of schizophrenia were elevated in black Caribbean (RR: 5.6; 95%CI: 3.4, 9.2; I^2^ = 0.77) and black African (RR: 4.7; 95% CI: 3.3, 6.8; I^2^ = 0.47) migrants and their descendants, compared with the baseline population. This pattern was also reported for the affective psychoses [10], [26*], [102*], [110*], including bipolar disorder [60], [73*], [86*], [100], [120*] and psychotic depression [72*], [73*] independently (see ON7). For substance-induced psychoses, one study reported higher first admission rates of cannabis-induced psychoses in black Caribbean men [102*], but unpublished data from the ÆSOP study [73*] suggested the near-opposite; 92.6% of people with a substance-induced psychosis were white British, with the remainder of mixed ethnicities.

Using available data on the relative risk of schizophrenia for people of black Caribbean ethnicity in England [26*], [73*], [85*], [86*], [93*], [117*] or by Caribbean birth [58*], [87*], compared with the baseline group used in these citations (white, white British or British-born), we inspected for possible publication bias, but found no evidence to support this (Egger's p = 0.70; see Figure S5).

The pattern of rates of psychotic disorder amongst Asian migrants and their offspring was less consistent [10], [26*], [58*], [72*], [73*], [93*]. The ÆSOP study [73*] did not report any significantly raised rates in people of Asian origin (n = 29), however data from an even smaller sample (n = 7) [93*] suggested rates of schizophrenia were elevated amongst Indian, Pakistani and other Asian groups (Figure 3) in comparison to the white population in North London. To date, the largest study date in these groups was conducted in East London [10], [26*], where 21.5% of the population at-risk were of Asian origin [10]. Rates of schizophrenia (n = 67) were notably elevated for Pakistani (IRR: 3.1; 95%CI: 1.2, 8.1) and Bangladeshi (IRR: 2.3; 95%CI: 1.1, 4.7) women [26], though not men, after adjustment for age, sex and socioeconomic status. For all clinically relevant psychoses, this effect was marginally stronger amongst first generation (RR: 3.6; 95%CI, 2.1, 6.4) than second-generation (RR: 2.3; 95%CI, 1.0, 5.3) Asian women [10]. Further inspection of the ÆSOP data also suggested there was weak support for the same sex-specific effect in the Asian group (IRR: 2.8; 95%CI: 0.9, 7.9) [73]. Two further citations [58*], [72*] reported elevated rates of schizophrenia for people born on the Indian Subcontinent [58*] and in India [72*], compared with those born in the UK. We pooled the available data on rates of schizophrenia in those of Asian ethnicities in England [26*], [73*], [93*], which yielded a RR of 2.4 (95%CI: 1.3, 4.5; I^2^ = 0.42) compared with the background white population. Where available, data for other psychotic disorders was mixed. Four citations did not find evidence for raised rates of affective psychoses in people of Asian birth or origin [10], [26*], [58*], [72*]. However, the ÆSOP study [73*] reported weak support for raised rates of bipolar disorder (IRR: 2.7; 95%CI: 0.9, 7.6) and psychotic depression (IRR: 3.0; 95%CI: 1.3, 7.1) in people of Asian origin, after adjustment for age and sex.

We identified fewer reports of incidence rates for other ethnic groups from the available literature. Three citations from two studies [10], [26*], [73*] reported an approximate doubling of risk of all clinically relevant psychoses in non-British white migrants, after adjustment for age and sex, though no clear pattern emerged in respect of specific disorders. One of these studies [73*] reported raised rates of psychotic disorders in those of mixed ethnicity, an effect which appeared to be highly pronounced for people of mixed white and black Caribbean ethnicity [26*], particularly with regard to affective psychotic syndromes (RR: 10.9; 95% CI: 4.5, 26.3). Estimates for other groups from further citations were highly heterogeneous [77*], [93*], [99*] (Figure 3).

In general, elevated rates of psychosis were not explained by age and sex differences between ethnic minority groups and the white/white British population [10], [26*], [73*], [80], [85*], [93*], [104], [117*]. We identified five citations which presented rates of psychotic disorder in different ethnic groups by age [52*], [73*], [85*], [86*], [102*], two of which simply dichotomized age at 30 years old as a proxy for migrant generation status [52*], [86*]. Those studies, along with a third citation [102*] which explicitly distinguished between first- and second-generation black Caribbean migrants, found broad support for raised rates of psychotic disorder for both generations. Two citations [73*], [85*] presented rates of psychotic disorder by ethnicity across several age groups; rates appeared elevated at all ages for black Caribbean [73*], [85*] and black African [73*] groups. One citation [10*] has recently extended the literature on psychosis risk by ethnicity and migrant status finding elevated rates of psychosis in several first- and second-generation ethnic minority groups. Data from the same study [26] also indicated that differences in rates between ethnic groups were not wholly explained by socioeconomic status, which only partially attenuated such associations.

### D. Incidence of psychotic disorders over time

We identified fifteen primary citations from nine studies which directly investigated possible changes in the incidence of psychotic disorders over time in England [14], [15], [46], [48], [53], [56], [60], [69], [70], [84], [95], [105], [106], [120], [123]. Median length of time over which rates were compared was 16 years, ranging from ten years (1996–2005) [123] to 114 years (1881–1994) [105]. Most citations investigated changes in incidence between the mid-1960 s and mid-1980 s [14], [53], [60], [70], [84], [106], [120] and were typically based on continuously-derived data from case registers [14], [53], [60], [70], [84], [120]. Two citations, based on data from the Mental Health Enquiry [15], [48], inspected changes in the incidence of psychotic disorder extending back to the 1950 s, while more recent data has largely been derived from repeated cross-sectional estimates [46], [56], [95], [105] or primary care [123].

The largest literature in England on rates over time is in relation to schizophrenia [14*], [46], [53*], [56], [60], [70*], [84], [95*], [105*], [106*]. Data were highly heterogeneous (Table 4) and no attempt to pool findings was made. Studies in London tended to report an increased rate of schizophrenia between 1965 and 1997 [14*], [46], [53*], [60], although these changes were possibly attributable to increases in the proportion of ethnic minority populations, currently at greater risk of psychoses (see above), living in the catchment area over the same time period. By contrast, available data from studies in Nottingham (Table 4) found no evidence of an increase in schizophrenia over roughly the same time period, with citations divided as to whether the rate had remained the same [84], [105*] or fallen [56], [95*]. In one citation [95*] this decline was matched by a corresponding increase in other non-affective disorders, such that the overall rate of non-affective psychoses had remained stable over time. Thus, genuine changes in the syndromal presentation of disorders [56], [70], the organization of mental health services [106] or shifts in diagnostic practice [95] over time might have accounted for reports of decline in schizophrenia incidence [56], [70*], [95*], [106*].

**Table 4:** Citations reporting incidence of schizophrenia over time in England, 1881–1999, organised by study setting.

Authors	Time period(s)	Setting	Contact type	Findings∧	Original authors' explanations
Allardyce *et al.* [46]	1979–841992–97	Camberwell, London (& Dumfries & Galloway†)	Case register & first contact	Increased rate in Camberwell over time, adjusted for age & sex (**+**)	Increase in ethnic minority population in Camberwell over time period. Rate in white group in 1992 was comparable between rural & urban settings
Boydell *et al.* [53]	1965–97	Camberwell, London	As above	As above (**+**)	Increase in ethnic minority population in Camberwell over time period.
Castle *et al.* [14], [59], [60]	1965–84	Camberwell, London	Case register	Trend towards increased rates (p = 0.06) (**+**)	As above
Harrison *et al.* [84]	1975–87	Nottingham	Case register	No change in rate (**∼**)	Changes elsewhere might be explained by migration
Kirkbride *et al.* [95]	1978–801992–941997–99	Nottingham	Case register + first onset	Decline in rate (**−**)	Diagnostic changes over time. Decline matched by corresponding increase in other non-affective psychoses. Overall, stable rates of non-affective psychosis
Brewin *et al.* [56]	1978–801992–94	Nottingham	Case register	Decline in rate (**−**)	Genuine change in the syndromal presentation of disorder
Nixon *et al.* [105] ‡	1881–19021978–801992–94	Nottingham	Case register + re-diagnosis of historical records	No change over 114 years (**∼**)	Stability of aetiologically-relevant social factors over time, though not across sociodemographic groups, may explain constant rate
de Alarcon *et al.* [70]	1975–86	Oxfordshire	First contact	Decline in rate (**−**)	Diagnostic changes over time, partially evidenced by increases in diagnosis of other “paranoid states” (i.e. other non-affective disorders)
Prince & Phelan [106]	1970–85	England	First admissions	Decline in rate (**−**)	Change of organisation of healthcare from inpatient to outpatient and possible population attitude shift in treatment of mentally ill may explain decline. Decline of schizophrenia set against parallel declines over same period for many types of mental illness. Argues against “true” decline (see [15])

&dagger; Results from Dumfries & Galloway (Scotland) not officially part of present review but included as part of study.

&Dagger; First time period lies outside the scope of this review, but results presented in table for completeness.

&and; (+) Increase in rate; (−) decrease in rate; (∼) no change in rate observed.

We identified two primary citations which had considered changes in the incidence of all psychotic disorders as a broad category over time [56], [95*], [123*]. One citation [95*] reported no evidence of changes in first contact rates (p = 0.19) over a twenty-year period (1978–80, 1992–94, 1997–99), using data from three methodologically similar studies in the same catchment area [95]. A second recent citation [123*], using primary care data, also failed to find evidence rates had changed over a ten-year period (1996–2005) [123].

Three citations were identified which reported incidence of affective psychoses over time [70*], [ 84*], [95*]. Two [70*], [84*] reported a decline in rates between 1975 and 1987, but this could be attributed to a change in the diagnostic classification of non-psychotic depression in ICD-9, which had previously been classified with the affective psychoses in ICD-8 [84]. This methodological issue was overcome in the third citation [95*] which demonstrated that the incidence of affective psychoses had remained predominately stable over three time periods between 1978 and 1999 (RR: 1.00; 95% CI: 0.98, 1.03). Data available for changes in the incidence of specific affective disorders were highly heterogeneous [48*], [60], [84*], [95*], [120*]. First admissions data in England and Wales between 1950 and 1960 suggested an increase in the hospitalized incidence of “manic depressive reaction” (bipolar disorder) but not “involutional melancholia” (depressive psychoses) [48*], however no formal statistical analyses were possible given limited published data. One further study [84*] attributed rises in first contact rates of mania between 1975 and 1986 to changes in diagnostic classification. A third study [60], [120*] found an apparent increase in schizomania among women, but not men, in South London, and no evidence for changes in the incidence of mania itself. Finally, first onset data from three time points in Nottingham [95*] suggested no change in the incidence of either bipolar disorder or the depressive psychoses over time.

We identified two citations which inspected incidence of substance-induced psychotic disorders over time [15*], [95*]. Der and colleagues [15*] reported no change in the admitted incidence of alcoholic psychoses in England and Wales between 1970–86. However, data from the other citation [95*] suggested that the incidence of all substance-induced psychoses had risen between 1979 and 1999 in Nottingham (RR per year: 1.15; 95% CI: 1.05, 1.25), after adjustment for age and sex; absolute incidence remained low (3.6 per 100 kpy; 95%CI: 1.9, 5.2).

To further inspect the possibility of changes in rates over time, we entered available data on the overall incidence of psychotic disorders (Tables 1a & 1b) into meta-regressions, where the mid-point of each study's case ascertainment period was entered as a covariate. This data suggested there was no evidence of change in the incidence of psychotic disorder over time (Table S1).

### E. Geographical variation in the incidence of psychotic disorders

Studies which considered geographical variation in the incidence of psychotic disorders in England were highly heterogeneous in diagnostic outcomes considered and methodological approach [1], [27], [54], [55], [62], [68], [78], [79], [83], [94], [96], [97], [100], [104], [121], [124]. Because there was evidence from the wider literature that non-affective psychoses, but not their affective counterparts, show geographical variation [7], [125], we did not report data for all clinically relevant psychoses here (but see full report, ON7).

We identified four citations [1*], [78*], [83*], [96*] which investigated the incidence of non-affective psychoses according to some metric of geographical variation. The earliest study [78*] inspected the distribution of non-affective psychoses in Nottingham between 1975 and 1980, observing higher rates in more socioeconomically deprived neighborhoods. The remaining two citations [1*], [96*] examined spatial variation in the incidence of non-affective psychoses in the ÆSOP study. One [1*] observed significantly higher rates in Southeast London compared with Nottingham (RR: 2.7; 95%CI: 2.2, 3.4) and Bristol (RR: 1.9; 95%CI: 2.7, 3.8), after adjustment for age and sex. The other [96*] reported rates of non-affective psychoses varied significantly between neighborhoods in Southeast London, after adjustment for age, sex and ethnicity. Finally, earlier data from Bristol also suggested an elevation in rates in inner-city neighborhoods (see below).

Most data on geographical variation in incidence was identified in relation to schizophrenia, both between [1*], [27*] and within cities [54*], [55*], [62*], [78], [79*], [94*], [97], [104]. In ÆSOP the inter-city differences described above persisted for schizophrenia [1*]. A further study reported no difference in incidence between two similarly-sized towns in Southern England, Chichester and Salisbury [27*]. The remaining citations inspected variation in incidence between smaller neighborhood units, typically demarcated by administrative boundaries. All studies observed some variation in the incidence of schizophrenia at the neighborhood level. In Nottingham [78], [79*] incidence rates were highest in inner-city areas characterized by greater levels of unemployment, rented accommodation and single persons. A later study of all clinically relevant psychoses in Nottingham [68*] also reported that rates of psychosis were elevated in the most deprived communities. However, in Bristol [83*] elevated rates of non-affective psychoses in the inner city correlated not to deprivation, but to the proportion of single persons in each community, something replicated in a more recent Dutch study [126], and consistent with the possibility that social isolation may be a marker for psychosis risk.

A body of citations were identified which investigated putative social factors associated with the incidence of schizophrenia [54*], [55*], [94*], [97] in Southeast London. While socioeconomic deprivation was weakly associated with the incidence of schizophrenia in the ÆSOP study [94*], [97], non-economic social factors were also reported to be related to the incidence of schizophrenia, after adjustment for age sex and ethnicity. These included lower levels of ethnic fragmentation (the extent to which people from the same ethnic group lived in concentrated residential patterns) and social cohesion (features related to the social organization of a neighborhood that, collectively, “facilitate coordination and cooperation for mutual benefit” [127] p.36) [94*], [97]. In the first study [97], the authors reported that rates of schizophrenia were significantly higher in neighborhoods with lower levels of social cohesion (indexed by voter turnout at local elections). In a follow-up [94*], which attempted to measure social cohesion more precisely using a separate cross-sectional household survey, the authors observed a non-linear association between social cohesion and schizophrenia incidence, such that rates were higher in neighborhoods which low and high levels of social cohesion, but not areas in the middle. The authors suggested that areas with *high* social cohesion could have had higher rates of schizophrenia if certain groups (such as minority ethnic groups) were prohibited from accessing social cohesion reported in these communities; there was some support for this in the data, where white groups were over-represented in the household social cohesion survey, and the u-shaped association with schizophrenia was also stronger for ethnic minority groups [94].

This effect is akin to the ethnic density effect (where schizophrenia risk increases amongst ethnic minority groups as they live in less ethnically dense communities with fewer people from similar ethnic backgrounds), for which there is also some independent support in the same dataset [94*], [97], from a separate study in South London [54*] and from a relevant citation published after the end of this review [128]. One earlier citation [62*], using all first admission data in England from the Mental Health Enquiry, found no evidence for such an effect. However, it considered ethnic density at national and regional levels, which may have been too broad to detect significant associations at smaller (i.e. neighborhood) levels. Finally, a further citation [55*] from South London considered whether role of socioeconomic inequality (*c.f.* absolute levels) was associated with the incidence of schizophrenia. Although there was not an overall effect of inequality, it was associated with higher rates of schizophrenia in the most deprived neighborhoods, suggesting interactive effects between absolute and relative deprivation.

There was less consistent evidence to support socio-spatial patterning of the affective psychoses [1*], [27*], [78], [79*], [83*], [96*], including bipolar disorder separately [100*], [121*]. De Alarcon *et al.* [27*] observed higher crude rates of affective psychoses in Chichester than Salisbury (RR: 1.28; 95%CI: 1.04, 1.58), while Wing and colleagues [121*] found higher crude rates of bipolar disorder in London than Salford, but neither study adjusted for potential confounders. The ÆSOP study also reported higher rates of affective psychoses [1*] and bipolar disorder [100*] in Southeast London compared with Nottingham or Bristol, having adjusted for age and sex, but these effects were smaller than for their non-affective counterparts and did not persist following additional control for ethnicity. Further, when rates of affective psychoses were compared within neighborhoods [96*], there was no evidence to support spatial variation in incidence, after adjustment for age, sex and ethnicity; these findings are consistent with the remaining literature identified by our review [78], [79*], [83*] and elsewhere [125], [129], [130], [131]. We did not identify any citations which had considered spatial variation in the incidence of depressive or substance-induced psychoses.

To supplement these studies we used random effects meta-regressions to consider whether the overall incidence of psychotic disorders (Tables 2 & Table 3) showed variation by urbanicity (Table S2). Our results suggested that greater urbanicity was associated with an increased crude incidence of both the non-affective psychoses (IRR: 1.022; 95%CI 1.017, 1.028; p<0.001) and schizophrenia (IRR: 1.03; 95%CI 1.01, 1.03; p = 0.01), but not affective or substance-induced psychoses.

### F. The effect of study quality on incidence rates

Overall there was little evidence that reported study quality had an effect on the incidence of psychotic disorders using the data identified during the course of this review, though for any single outcome mean study quality was generally high (Table S3). Using random effects meta-regressions we identified one outcome (depressive psychoses) which showed an association with study quality, with higher quality studies tending to report significantly lower crude rates of disorder (IRR: 0.81; 95%CI: 0.71, 0.93).

## Discussion

We have conducted the largest systematic review of the incidence and epidemiology of schizophrenia and other psychotic disorders in England. We have developed and implemented a thorough, systematic research strategy to identify all citations reporting original incidence data on seven clinically relevant psychotic outcomes. For each of these, we have delineated overall incidence in England since 1950, and using detailed descriptive and novel statistical analyses we have identified key domains of variation. This approach confirmed differences in rates by age and gender, ethnicity and migration, and also revealed differences in rates by place and neighborhood-level socio-environmental factors, including ethnic density, social fragmentation and socioeconomic inequality. By contrast, there was little evidence of overall changes in the incidence of psychotic disorder over time in England or according to reported study quality.

### A. Principal findings

The pooled incidence of psychotic disorders in England (Figure S2) were broadly in keeping with findings from the wider psychiatric epidemiology literature [2]. Our results lend credence to the methodological rigor of both our review, and, generally, the individual studies which reported original data; as would be expected, highest incidence rates were identified for all clinically relevant psychoses, followed by non-affective psychoses and schizophrenia. Relative to non-affective psychoses, the incidence of their affective counterparts was of an order of magnitude lower; though the prevalence of all psychotic disorders in England and elsewhere continues to present substantial psychiatric morbidity [132]. The incidence of specifically diagnosed substance-induced disorders was generally rare, but substance *misuse* in the context of ongoing psychotic disorder remains a serious public health challenge in terms of poor outcome and high service use [133], [134].

These figures, of course, belie considerable heterogeneity in incidence rates, both by specific outcome and sociodemographic group. This heterogeneity emerges as the primary finding from our systematic review. With respect to age and gender, there was broad support for the typically-observed incidence of non-affective psychoses [9], with peak incidence for men and women in their twenties, declining thereafter for both sexes with a smaller, secondary peak in incidence for women from midlife. For non-affective psychoses, but not their affective counterparts, rates were generally elevated amongst men prior to midlife. Using a novel application of random effects fractional polynomial meta-regression on the available data [36], we were able to empirically confirm this interaction in a meta-analytical framework for the first time for all clinically relevant psychoses, non-affective psychoses, schizophrenia and the affective psychoses.

Our review identified raised rates of psychotic disorders across several ethnic minority groups. Effects were strongest, and most consistent, amongst migrants and their descendants of black Caribbean and black African origin. Although the evidence in England for raised rates amongst ethnic minority groups descendant from the Indian subcontinent has been interpreted as equivocal, our review suggested some elevation in rates for this group (pooled OR: 2.4; 95% CI: 1.3, 4.5), a phenomenon potentially restricted to women [26], [73]. There was emerging evidence of raised rates amongst people of mixed ethnicity, a possible marker of ‘third-generation’ descendants, and some suggestion of a smaller, though significant elevation in rates amongst non-British white migrant groups. Generally, these effects were reported in separate settings, for several outcomes (with the exception of substance-induced disorders), and after control for putative confounders (including socioeconomic status [26]) and improvements in study design over time, including more precise case and denominator estimation [10], [26], [73], [117], consensus diagnosis by a multicultural panel of psychiatrists [73], partial blinding to ethnicity during the diagnostic process [73] and standardization of diagnostic criteria [10], [26], [73], [117].

There was little direct evidence to support genuine changes in the incidence of psychotic disorders over time [95], [123], which might have otherwise indicated a change in the frequency of exposure to, or impact of candidate social or drug-related risk factors for psychosis. One study suggested an increase in the incidence of substance-induced psychoses, but this change did not affect the overall incidence of first episode psychosis reported in that study over time. Meta-regression of crude incidence rates over time from independent reports also supported this. Reports of changes in the incidence of specific disorders over time were possibly attributable (often by the original authors) to changes in the underlying population at-risk, revisions in diagnostic classifications, changing diagnostic fashion and re-organization of mental health service provision during the 1980 s and 1990 s. Such explanations are in accordance with the wider, international literature [135], [136], [137], [138]. There were, however, few studies.

Studies which addressed geographical variation in incidence rates were diverse in location, methodology, exposure of interest and disorder studied. The strongest evidence for a geographical gradient in incidence was for non-affective psychoses, including schizophrenia, with somewhat equivocal evidence for their affective counterparts. Studies conducted in London, England's most urban conurbation, consistently reported the highest overall incidence of non-affective psychoses and schizophrenia. This was confirmed in our meta-regression which revealed a significant linear association between these disorders and our urbanicity index. This variation was reported to be independent of differences in the age, sex and ethnic population structure of different geographical areas, and correlated to a number of socio-environmental factors including ethnic density, social cohesion, social fragmentation, deprivation and inequality. By contrast, for the affective psychoses, including bipolar disorder and the depressive psychoses, meta-regression did not reveal any association between incidence rates and urbanicity.

### B. Meaning of findings

Our findings in regard to the incidence of psychotic disorders by age and gender are consistent with the wider international literature [2], [9], [139], and may implicate a biological component to disorder. Although this hormone also appears to be associated with psychopathology [140], and there is an increased risk of psychosis in women at other times of estrogen depletion such as immediately after birth, the latest Cochrane Review did not find enough evidence to promote its use as an intervention [141]. Furthermore, since the general pattern in men and women from the mid-twenties until menopause is a decline in incidence, other factors which change as a function of age are also implicated in psychosis etiology. Given that the menopause is a bio-psycho-socio-cultural experience, apparent explanations for a secondary peak onset of psychosis in women at this time could be both biological and sociocultural in origin, and, tentatively, may include the loss of a potentially protective role for estrogen, changes in reactivity to dopamine and/or increased social stress for some women resulting from changes in identity and status.

A change in the incidence of psychotic disorders over time would implicate a change in the underlying prevalence of one or more exposures, given relatively fixed genetics over the short term (i.e. over the 60 years covered by this review). Given the strong genetic component likely to underpin psychosis risk [142] it is perhaps unsurprising rates in England appear unlikely to have changed markedly since at least 1950, having acknowledged compositional changes to the underlying population. This, of course, does not preclude an additional (socio-)environmental component to the etiology of these disorders, but it implies that exposure to these factors, such as deprivation, social isolation or traumatic life events has remained – on average – relatively constant over time. However, we also note a more complex explanation may explain the apparent stability of rates over time; improved prenatal and obstetric care may have reduced psychosis incidence (in offspring) on the one hand, coupled with increases in cannabis use or reductions in the levels of social cohesion may have acted in a compensatory way to increase incidence on the other, overall leaving the impression of stable rates. It is also possible that cumulative and (or) interactive environmental risk factors might need to reach a threshold before being translated into an effect on incidence. This is likely to have occurred in specific areas or within specific minority groups, but not in England as a whole. Interestingly, the only disorders which showed any discernible increase over time in this review were substance-induced psychoses [95]. This is relevant here given dramatic changes in substance abuse over the same time period [133], [134], [143], and the likely causal association between cannabis and psychosis [144], [145], [146]. Continued surveillance of the incidence of psychotic disorders is vital [25], [147], [148], given that model projections suggest any link between cannabis use and psychosis will begin to translate into tangible changes in incidence over the next decade [17]. We did not identify any English study which had directly considered the role of substance use on the incidence of psychotic disorders, principally because of a lack of corresponding denominator data on substance use in the general population necessary to estimate incidence rates. Longitudinal monitoring of the underlying prevalence of socio-environmental risk factors for psychosis may shed light on explanations for any (or lack of) temporal changes in incidence.

We next consider the meaning of findings in relation to ethnicity and geographical location. We initially draw upon relevant literature in regard to ethnicity, but go on to show that there is likely to be a degree of synergy between the suite of risk factors which putatively account for raised psychosis rates amongst migrants and their offspring and for people born, growing up and living in urban environments.

Raised rates of psychotic disorder in ethnic minority groups are one of the most frequently replicated and yet still controversial public health challenges in contemporary psychiatric epidemiology [149], [150], [151], [152]. Such observations are not new [87], [153], [154], are not a phenomenon limited to the UK or even Europe [153], [154], [155], [156], and are not limited to people of black ethnicity [10], [153], [154], [157]. Nevertheless, not all migrant groups in England [52], [73] or elsewhere [158], [159] exhibit the same risk profile, with considerable variation by ethnicity, sex and other socio-environmental factors. Such heterogeneity is likely to reveal clues to the possible determinants of psychosis incidence according to ethnicity.

In England this discussion has centered on the controversial but consistently raised rates of psychotic disorders in people of black Caribbean and African origin. These populations largely reflect patterns of migration to the UK following World War II, which saw substantial labor-related immigration from former colonial regions, including the Caribbean and the Indian subcontinent (as well as Gujarati Indians from Uganda). Migration from Africa had both earlier origins (resulting from Britain's involvement in slavery) and more recent origins, particularly during the 1990 s. A number of early hypotheses focused on the possibility that first generation migrants were more likely to be predisposed to psychosis, though there is now strong evidence against this (see Box S2). This includes a well-designed thought experiment disproving selective migration as an explanation of raised rates in Surinamese migrants to the Netherlands [160], raised rates in so-called second generation groups [10] and the complexity of migration as a task when weighed against cognitive impairment often experienced in the prodromal phase of psychosis [161]. There is no evidence to suggest that rates of psychosis in Jamaica [162], Trinidad and Tobago [163] or Barbados [164] are higher than the rate in the white British population, though we note a current lack of corresponding incidence studies in other relevant settings, including the Indian Subcontinent and sub-Saharan Africa.

Misdiagnosis has often been cited as a potential explanation of higher rates in ethnic minority groups living in England (Box S2). Evidence for this hypothesis remains weak. Institutionalized racism in health and other public services presents an important challenge to deliver culturally, religiously and ethnically sensitive services [150], and psychiatry has been no stranger to this problem [165]. Nevertheless, one study found that while both a Jamaican and British psychiatrist performed poorly when diagnosing schizophrenia in a series of case vignettes, a racial bias did not explain this difference [166]. The use of standardized diagnoses and partial blinding of a multi-ethnic panel of diagnosticians to the ethnicity of cases in contemporary incidence studies of psychosis further argue against misdiagnosis as the sole explanation of this phenomenon. The possible medicalization of culturally-bound behaviors as psychotic by those trained under a Western medicine paradigm has been less fully explored, but new studies in low and middle income countries will offer tantalizing opportunities for cross-cultural validation studies. One issue that we were unable to address in this data was whether raised rates of schizophrenia in black and minority ethnic groups could be instead attributed to misclassification of acute and transient psychoses, which may sometimes resemble schizophrenic symptoms at presentation and may be more common in certain ethnic minority groups [167]. While diagnosis is often difficult at first presentation, we do not believe this would offer an adequate explanation of raised rates in such groups since rates of other psychotic disorders, including bipolar disorder and psychotic depression have also been shown to be raised in ethnic minority groups at first presentation. Furthermore, recent studies use standardized diagnostic criteria, blind to ethnicity, making this misdiagnosis even less likely. Given the available literature on psychotic disorders in minority ethnic groups [11], we would thus expect a preponderance of acute and transient psychoses *in addition* to schizophrenia and other psychotic disorders, not instead of them. Either way, this excess still clearly presents a major public health concern [151]. Overall, we believe that the issue of institutionalized racism should be distinguished from the compelling international evidence that many migrants and their descendants, with the caveat of variation as noted above, experience genuinely raised levels of psychosis compared with the majority ethnic group in a particular locale [11]. This issue should be seen as real, demanding sensitive mental health service provision and ongoing public health attention [151].

Excess rates in ethnic minority groups are not confounded by age and sex [10], [73], [117], and a further recent study found rates were only partially attenuated by additional control for socioeconomic status [26]. However, a suite of other, complex socio-cultural and socio-environmental experiences may be relevant to understanding variation in rates of psychotic disorders according to ethnicity. These might include both post-migratory experiences (see below) and the migration process itself, which will require a degree of social competency to overcome logistical, political and economic barriers in order to manage a successful migration. This process may lead to considerable social stress for some individuals, perhaps increasing psychosis risk.

Post-migratory experiences, or experiences related to minority ethnic group membership, may also be relevant to variation in rates of psychosis. For example, an ecological study in the Netherlands reported that ethnic groups which perceived greater levels of discrimination also experienced higher rates of psychotic disorder [168], although initial findings at the individual level failed to replicate this association [169]. Further work from the same group reported an association between stronger negative ethnic identity and the odds of psychotic disorder [170]. This finding resonates with recent reports of elevated psychosis rates for people of mixed ethnicity in England [26], [73] and rates of non-affective psychosis also appear to be higher in neighborhoods with greater levels of ethnic fragmentation [94], and, independently, for ethnic minority groups when they make up a smaller proportion of the overall neighborhood population [54], [94], [125], [128], [171]. These factors might putatively influence psychosis risk through social stress [172], [173], in two distinct, but simultaneous processes.

First, those with more negative ethnic identity, or who live apart from others who share similar sociocultural experiences, migration histories, values, beliefs, attitudes and lifestyles may lack the social capital required to successfully mitigate the challenges encountered as a first generation migrant and/or member of an ethnic minority group. This lack of social capital may make it harder to enter local labor markets or develop social support networks to protect against both non-racial and racial social stressors. In the overall population at-risk, measures which index social capital, including social cohesion [94], [97], residential mobility (population instability) [174] and social fragmentation [175], [176] are all associated with higher rates of psychosis, and may link into higher rates of psychosis observed with urban birth [7], [177], upbringing [178] and living [1], [124], [179], [180]. Nevertheless, the above association between ethnic identity and psychosis is not fully understood [170]; one study in England reported an association between *positive* ethnic identity and the odds of psychosis [181], in opposition to the Dutch finding.

A second possibility is that people from ethnic minority groups who live in less ethnically dense neighborhoods may be exposed to greater levels of social stress not only because they may have fewer resources available which would otherwise confer protection against such stress, but because the prevalence of exposure to those stressors, such as experiences of racism, is greater in such communities. Findings in regard to psychosis risk and the stronger u-shaped relationship with social cohesion in black and minority ethnic groups (where response to the social cohesion survey was over-representative of people from white ethnicities) provides some support for this assertion [94]. Further reports from the ÆSOP study, using the available case-control data, have revealed direct evidence at the individual level of differences in the prevalence of exposure to social stressors associated with psychosis between ethnic groups [182], [183]. These findings potentially support this hypothesis. In these reports, it was observed that both parental separation/death and markers of social disadvantage were independently and significantly associated with greater risk of schizophrenia in the black Caribbean and white British groups. However, the prevalence of each of these factors was significantly greater amongst black Caribbean cases and controls [182], [183].

If, as data at the neighborhood-level is consistent with, social support is protective against psychosis, it should follow that adverse life events at the individual level impact negatively on psychosis risk. Data from several studies now bear this out. Adverse life events in childhood seem to be particularly pervasive on later psychosis risk. For example, lower socioeconomic position during childhood has been found to be associated with greater psychosis risk in a large Swedish population sample [184]. Traumatic events in childhood, including physical and sexual abuse [185], [186], parental death [183], separation from a parent [183] and institutionalized care and victimization [185] have all been associated with an increased odds of experiencing psychosis. Furthermore, severity of abuse experienced appears to increase psychosis risk in a dose-response fashion [187].

### C. Methodological considerations

To our knowledge our series of systematic reviews are the most comprehensive attempt to characterize the epidemiology of psychotic disorders ever conducted in England. We have adhered to many principles of the Cochrane Collaboration in order to develop a thorough and exhaustive search strategy and data extraction system standardized across the reviews in this series. We formed a multidisciplinary team of content-area experts, librarians, specialist systematic reviews and statisticians with expertise in meta-statistics to ensure the review was conducted to the highest possible standards. The quality of our approach was assured in several phases; during initial planning we consulted international content-area experts with experience in systematic reviews [2]. Our review underwent peer-review during the bidding stage following the funder's call for proposals and, again, prior to the publication of the full, final report (ON7). Both stages of peer-review, in addition to those necessary for academic publication, have improved the design, conduct and dissemination of our findings.

We are confident we identified all relevant published, grey and unpublished literature through our broad, multistage search strategy. Where we were able to inspect possible bias resulting from publication or study design (overall incidence of schizophrenia, and differences in rates between the black Caribbean group and the baseline population), we found no evidence to support this, caveated by the fact that formal tests, such as Egger's [38], have less power to detect such effects when between-study heterogeneity is marked. We acknowledge that limiting the geographical scope of this review to studies conducted wholly or partially in England represents a limitation in terms of generalisability to other settings. Nevertheless, we have highlighted those findings which appear to hold across international settings (age, sex, time) and those which generally hold across international settings but are also context dependent (ethnicity and urbanicity). Such findings may provide important etiological clues to our understanding of psychotic disorders.

While our search strategies and data extraction were comprehensive, we have not been exhaustive in reporting every possible analysis delineated in our citation matrix (ON6). Instead, here, we have reported the findings most pertinent to our understanding of the incidence and associated socio-demographic and socioenvironmental risk factors for psychoses. The open design of our review, together with the raw data which we have made available to the academic community, permits further analyses of given areas of interest. Furthermore, we have designed our review to be updatable such that the search strategy can be easily extended into more recent time periods to form a dynamic, durable resource for the academic community.

We did not identify any incidence study which had attempted to investigate whether aspects of the physical or built environment were associated with the incidence of psychotic disorders. A handful of studies reported an excess of psychotic disorders amongst those born in winter months [179], [188], [189], consistent with viral or nutritional hypotheses for psychosis [190], [191], [192], [193], but these citations did not meet criteria for this review [179], [188], [189].

One important limitation of the incidence literature in England is that studies are predominantly cross-sectional in design. While a number of associations have been reported in this review, determining their likely causality remains a challenge. Rather than dismissing these reports, it is preferable to consider them alongside the available findings from other study designs and settings, including birth cohorts [194], other longitudinal studies [176], [178] (including those which utilize national population registers [7], [177]), case-control studies [182], [183], [186] and emerging neuroepidemiological imaging studies [173], which broadly support a relationship between early life stressors, socioenvironmental exposures and psychosis risk. While we acknowledge the possibility that social drift may explain a degree of the association between incidence and urbanicity [195], social causation and drift are not necessarily mutually exclusive. An untested possibility is that one generates the other, leading vulnerable individuals to a perpetuating cascade of deleterious life events and social stressors which result in the manifestation of psychosis, potentially through mechanisms such as aberrant salience [172].

The conclusions of our review clearly depend on the comprehensiveness of case enumeration (i.e. the numerator), and accuracy of studies conducted in English catchment areas over the period (1950–2009). In this regard, we had to make some assumptions to enable inter-study comparisons. First, we recognize that age at first presentation, contact or hospitalization are not necessarily indicative of age at first onset, and that a small number of people will experience several months or even years of untreated *psychosis* [196]. Second, disorder when measured by other endophenotypic markers, such as cognitive decline or social withdrawal, may have given rise to alternative incidence patterns than those typically reported; here duration of untreated *illness* becomes relevant [197], although we note a dearth of incidence studies which have incorporated this concept. Further, we recognize that studies included in this report adopted different definitions of age (first presentation/contact/hospitalization) across which we assumed some commonality. We reasoned this was a pragmatic assumption given that definitions of incident age often reflected dominant models of mental healthcare at the time the study was conducted (i.e. “hospitalization” was typically adopted by studies prior to mental healthcare's devolution to “care in the community”, when inpatient services presented the main source of mental healthcare for people experiencing psychosis). Although differing definitions could have affected patterns of incidence, the findings presented here (for example, in respect of age, sex and ethnicity) were generally consistent, irrespective of the underlying definition of incidence adopted in each study.

Similarly, the strength of our findings also depends on the reliability of enumeration of denominator data upon which incidence rates were derived. For example, differential under-enumeration of particular subgroups, such as young men or ethnic minority groups, in the denominator could lead to observations of artificially raised rates for such groups. We do not believe this would be sufficient to entirely explain our findings (for example, of raised rates in ethnic minority groups), because: (a) while the 1991 Census was known to under-enumerate certain strata (particularly young men and ethnic minority groups), the Office for National Statistics [ONS] published correction factors to adjust raw denominator data [198], and adjustment for these did not substantially alter observed findings included in this review [117]; (b) the 2001 census was designed *a priori* to minimize such under-enumeration [199], and the pattern of variation in rates derived using this denominator source have remained largely unaltered [10], [26], [73]; (c) raised rates in some groups were large in comparison with the white British group, meaning underestimation would have had to have been substantial to fully explain the findings (for example in the ÆSOP study we estimate that between 500% and 600% of the enumerated black Caribbean population would have had to have been missed by the 2001 census in order to achieve parity of incidence between this group and the white British population for all psychotic disorders; under-enumeration estimates for this group by ONS were typically placed between 1–16% [198]).

In order to minimize the potential for publication bias to affect our results we rated the reported methodological quality of each citation and found little evidence to suggest reported rates were affected by study quality. Our measure had some psychometric validity (ON3), though we acknowledge that *reported* study quality may not be perfectly correlated with *actual* study quality. We assumed that reporting such features was equivalent to their conduct, and reasoned that a failure to report such facets, where they had been conducted, could itself be regarded as a marker of poorer quality.

Our application of meta-analysis was fairly conventional, but we urge caution in the interpretation of any pooled estimates in favor of acknowledging heterogeneity in rates. The I^2^-statistics we reported were generally large, particularly for overall crude rates of psychosis. While we cannot exclude the possibility that some of this variation is stochastic, the data we have presented here suggest much of this may be due to exposure to risk factors according to age, sex, ethnicity and urban living. Our use of meta-regression analyses, including fractional polynomial regression by age and sex, is more novel. These procedures are only just becoming established in the biostatistical literature [33], [36], [40], [44], [200], but we have no reason to question their applicability or implementation. One key variable used in our meta-regressions was our urbanicity index. Here we asked five British content-area experts to rank study settings in terms of perceived urbanicity, a subjective rating. We took this unique, novel approach because it was not possible to obtain more objective measures of urbanicity in many citations, where either the catchment area had not been precisely defined or where it would have been difficult to retrospectively estimate an objective measure of urbanicity (such as population density). The stability of this ranking over several raters enhanced the reliability and validity of this measure, though we cannot exclude the possibility that bias could arise. We only used this meta-regression to supplement our systematic review of the literature already pertaining to geographical variance in rates. Interestingly, our meta-regression approach supported these findings, offering some validity to our ranking.

### D. Conclusion

We have demonstrated considerable heterogeneity in the incidence of psychotic disorders in England over the last 50 years. Overall, the findings support the wider research literature that the epidemiological landscape is rich with contours and gradients [122], which have potentially important implications for both health service planning and our etiological understanding of psychotic disorders. While we condone clinical services acknowledging all individual diversity, the data suggest that commissioners need to take some of these factors into account, particularly with regard to age, sex and ethnicity, when planning services. We recommend that our work is taken further in terms of developing practical prediction tools for those providing mental health services; this would now be a relatively simple step, particularly given the replicability of findings in regard to age, sex and ethnicity, together with their stability over time. We are currently developing such models using available empirical data. Valid models are critical for accurate, efficient health service planning. This issue has been highlighted by reports of underestimation of anticipated service use when commissioning new mental health services [16], [201].

## Supporting Information

Figure S1 Citation matrix conceptualizing research streams, themes & blocks to which citations might contribute original data in our series of systematic reviews. A “research stream” is defined as a broad population group covered by our series of systematic reviews. Here, we focus on incidence studies in the general adult population. A “research theme” is the diagnostic outcome under consideration (see “Diagnostic Outcomes” in Methods). Other non-affective [NA] psychoses are not included as a separate category of analysis. Finally, a “research block” represents the main groups of “risk factors” by which citations will be systematically reviewed. “Other” risk factors are included in review but too heterogeneous to list all here. ^1^Study filters will be applied to research stream, theme & block permutations relevant to specific review aims & objectives. As the level of specialization (right to left) and focus (top to bottom) increases we expect the yield of studies relevant to the systematic review objective under analysis to decrease.
(DOCX)

Figure S2 Pooled incidence rates of psychotic disorders by diagnostic category.
(DOCX)

Figure S3 Funnel plot of log incidence rates of schizophrenia in relation to study size. There is little evidence of publication bias in citations of the incidence of schizophrenia when log incidence is plotted against each study's standard error (i.e. sample size). This was consistent with Egger's test of bias which found no evidence of bias (p = 0.24), though between-study heterogeneity (I^2^ = 0.97) may weaken power to detect bias [37].
(DOCX)

Figure S4 Incidence of schizophrenia by age and gender in England, 1950–2009, pooled and per relevant citation. The thin solid and dashed lines present rates of schizophrenia from individual studies for men and women, respectively. Thick solid lines present the unweighted mean rate for each strata, from these studies. Unweighted means are preferred in this instance because no model assumption underpins the data. Further, in the context of a random effects meta-analysis, the weighted mean approximates the unweighted mean as heterogeneity becomes large, as is clearly evident here.
(DOCX)

Figure S5 Funnel plot of log relative risk of schizophrenia in black Caribbean migrants and their offspring compared with the baseline population, by study size. This funnel plot shows little evidence of publication bias in citations where the relative risk of schizophrenia in the black Caribbean group could be estimated in relation to the baseline population, when log relative risk is plotted against each study's standard error (i.e. sample size). This was consistent with Egger's test of bias which found no evidence of bias (p = 0.70), though between-study heterogeneity (I^2^ = 0.77) may weaken power to detect bias, and caution is recommended [37]. The baseline group was either the white, white British or non-Caribbean born group as per original study.
(DOCX)

Table S1 Meta-regression to investigate changes in the incidence of psychotic disorders in England over time. We conducted random effects meta-regressions on available data on the overall crude incidence of various psychotic disorders to investigate whether there was any evidence to support a change in the incidence of disorders over time. Overall, there was little evidence from meta-regressions to support this possibility.
(DOCX)

Table S2 Meta-regression to investigate changes in the incidence of psychotic disorders in England by urbanicity. We conducted random effects meta-regressions on available data on the overall crude incidence of various psychotic disorders to investigate whether there was any evidence to support a change in the incidence of disorders according to our measure of urbanicity. The table shows there was some support for an increasing crude incidence of non-affective psychotic disorders (including schizophrenia) with increased urbanicity, but not for other disorders including the affective psychosis and substance-induced psychotic disorders.
(DOCX)

Table S3 Meta-regression to investigate changes in the incidence of psychotic disorders in England by study quality. We conducted random effects meta-regressions on available data on the overall crude incidence of various psychotic disorders to investigate whether there was any evidence to support a change in the incidence of disorders according to study quality. Overall there was little evidence to support this, although we noted that higher quality studies tended to report a lower incidence of the depressive psychoses.
(DOCX)

Box S1 Description of study quality criterion. Each citation included in this review was rated by the authors (JBK, AE) according to seven study quality criterion we reasoned that, if reported, could be taken to indicate methodological rigor. Details of each criterion are provided here.
(DOCX)

Box S2 Principle hypotheses to explain raised rates of psychotic disorder in migrant groups and their offspring. Here we summarize the main hypotheses that have been proposed to explain the excess incidence of psychotic disorders in migrants and their offspring. For each, we provide a description of the hypothesis, who originally proposed it and the evidence for and against.
(DOCX)

Appendix S1 PRISMA checklist. Details of how this systematic review conformed to the PRISMA standards for systematic reviewing.
(DOCX)
