# Supplementary material for: Incidence of Schizophrenia and Other Psychoses in England, 1950–2009: A Systematic Review and Meta-Analyses
Source: PLoS One. 2012 Mar 22;7(3):e31660. doi: 10.1371/journal.pone.0031660 (PMC3310436; doi:10.1371/journal.pone.0031660)
Supplement: Figure S1 — Citation matrix conceptualizing research streams, themes & blocks to which citations might contribute original data in our series of systematic reviews. A “research stream” is defined as a broad population group covered by our series of systematic reviews. Here, we focus on incidence studies in the general adult population. A “research theme” is the diagnostic outcome under consideration (see “Diagnostic Outcomes” in Methods). Other non-affective [NA] psychoses are not included as a separate category of analysis. Finally, a “research block” represents the main groups of “risk factors” by which citations will be systematically reviewed. “Other” risk factors are included in review but too heterogeneous to list all here. 1Study filters will be applied to research stream, theme & block permutations relevant to specific review aims & objectives. As the level of specialization (right to left) and focus (top to bottom) increases we expect the yield of studies relevant to the systematic review objective under analysis to decrease. (DOCX) [file pone.0031660.s001.docx]

| **Figure S1: Citation matrix conceptualising research streams, themes & blocks to which citations might contribute original data in our series of systematic reviews** |
| --- |

ETHNCITY x SEX, ETHNICITY x AGE, ETC…

**ALL PSYCHOTIC DISORDERS**

SCHIZOPHRENIA

NON-AFFECTIVE PSYCHOSES

AFFECTIVE PSYCHOSES

SUBSTANCE-INDUCED PSYCHOSES

BIPOLAR DISORDER

DEPRESSIVE PSYCHOSES

OTHER NA PSYCHOSES

**RESEARCH THEME**

PREVALENCE

POINT

PERIOD

**RESEARCH STREAM**

INCIDENCE

PREVALENCE

**GENERAL ADULT POPULATION**

**SPECIALIST POPULATIONS**

RISK FACTORS

INCIDENCE

RISK FACTORS

POINT

PERIOD

LIFETIME

LIFETIME

**RESEARCH BLOCK**

**OVERALL RATES**

TIME

AGE

SEX

ETHNICITY

COUNTRY OF BIRTH

GEOGRAPHY

URBANICITY

PLACE

ETHNIC DENSITY

AGE vs. SEX

OTHER

Decreasing size of expected yield^1^

*Degree of specialisation*

*Degree of focus*

***Research stream*:** broad population group covered by our series of systematic reviews. Here, we focus on incidence studies in the general adult population.

***Research theme*:** diagnostic outcome under consideration (see “Diagnostic Outcomes” in Methods). Other non-affective [NA] psychoses are not included as a separate category of analysis.

***Research block*:** The main “risk factors” by which research will be systematically reviewed. “Other” risk factors are included in review but too heterogeneous to list all here.

^1^Study filters will be applied to research stream, theme & block permutations relevant to specific review aims & objectives. As the level of specialisation (right to left) and focus (top to bottom) increases we expect the yield of studies relevant to the systematic review objective under analysis to decrease.
