# Supplementary material for: Incidence of Schizophrenia and Other Psychoses in England, 1950–2009: A Systematic Review and Meta-Analyses
Source: PLoS One. 2012 Mar 22;7(3):e31660. doi: 10.1371/journal.pone.0031660 (PMC3310436; doi:10.1371/journal.pone.0031660)
Supplement: Figure S2 — Pooled incidence rates of psychotic disorders by diagnostic category. (DOCX) [file pone.0031660.s002.docx]

| **Figure S2: Pooled incidence rates of psychotic disorders by diagnostic category** |
| --- |
