# Supplementary figures and images for: Incidence of Schizophrenia and Other Psychoses in England, 1950–2009: A Systematic Review and Meta-Analyses
Source: PLoS One. 2012 Mar 22;7(3):e31660. doi: 10.1371/journal.pone.0031660 (PMC3310436; doi:10.1371/journal.pone.0031660)

**Figure S3: Funnel plot of log incidence rates of schizophrenia in relation to study size
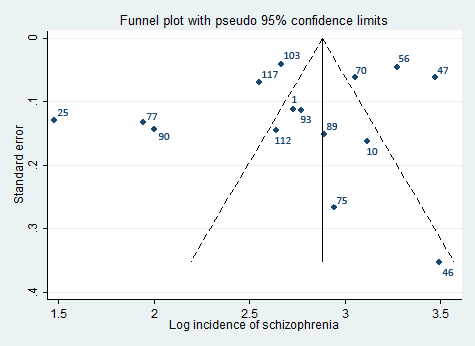
**

Supplement: Figure S3 — Funnel plot of log incidence rates of schizophrenia in relation to study size. There is little evidence of publication bias in citations of the incidence of schizophrenia when log incidence is plotted against each study's standard error (i.e. sample size). This was consistent with Egger's test of bias which found no evidence of bias (p = 0.24), though between-study heterogeneity (I2 = 0.97) may weaken power to detect bias [37]. (DOCX) [file pone.0031660.s003.docx]
