# Supplementary material for: Incidence of Schizophrenia and Other Psychoses in England, 1950–2009: A Systematic Review and Meta-Analyses
Source: PLoS One. 2012 Mar 22;7(3):e31660. doi: 10.1371/journal.pone.0031660 (PMC3310436; doi:10.1371/journal.pone.0031660)

**Figure S4: Incidence of schizophrenia by age and gender in England, 1950-2009, pooled and per relevant citation**


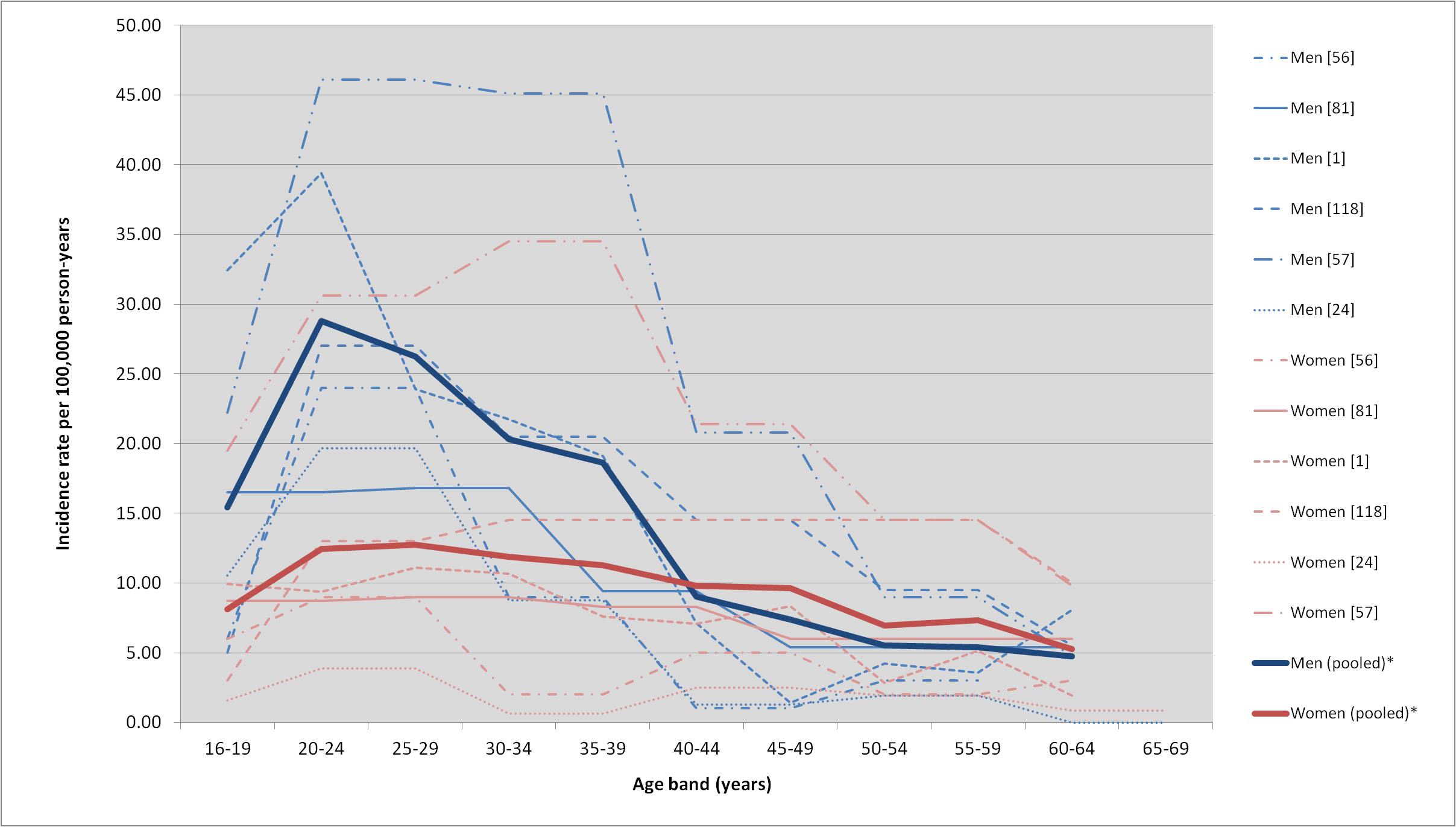

Supplement: Figure S4 — Incidence of schizophrenia by age and gender in England, 1950–2009, pooled and per relevant citation. The thin solid and dashed lines present rates of schizophrenia from individual studies for men and women, respectively. Thick solid lines present the unweighted mean rate for each strata, from these studies. Unweighted means are preferred in this instance because no model assumption underpins the data. Further, in the context of a random effects meta-analysis, the weighted mean approximates the unweighted mean as heterogeneity becomes large, as is clearly evident here. (DOCX) [file pone.0031660.s004.docx]
