# Supplementary material for: Incidence of Schizophrenia and Other Psychoses in England, 1950–2009: A Systematic Review and Meta-Analyses
Source: PLoS One. 2012 Mar 22;7(3):e31660. doi: 10.1371/journal.pone.0031660 (PMC3310436; doi:10.1371/journal.pone.0031660)
Supplement: Figure S5 — Funnel plot of log relative risk of schizophrenia in black Caribbean migrants and their offspring compared with the baseline population, by study size. This funnel plot shows little evidence of publication bias in citations where the relative risk of schizophrenia in the black Caribbean group could be estimated in relation to the baseline population, when log relative risk is plotted against each study's standard error (i.e. sample size). This was consistent with Egger's test of bias which found no evidence of bias (p = 0.70), though between-study heterogeneity (I2 = 0.77) may weaken power to detect bias, and caution is recommended [37]. The baseline group was either the white, white British or non-Caribbean born group as per original study. (DOCX) [file pone.0031660.s005.docx]

**
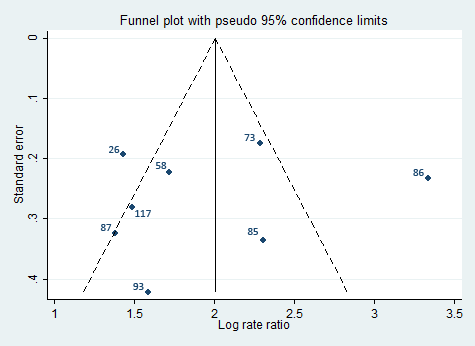
Figure S5: Funnel plot of log relative risk of schizophrenia in black Caribbean migrants and their offspring compared with the baseline population,* by study size**
