# Supplementary material for: Incidence of Schizophrenia and Other Psychoses in England, 1950–2009: A Systematic Review and Meta-Analyses
Source: PLoS One. 2012 Mar 22;7(3):e31660. doi: 10.1371/journal.pone.0031660 (PMC3310436; doi:10.1371/journal.pone.0031660)
Supplement: Table S1 — Meta-regression to investigate changes in the incidence of psychotic disorders in England over time. We conducted random effects meta-regressions on available data on the overall crude incidence of various psychotic disorders to investigate whether there was any evidence to support a change in the incidence of disorders over time. Overall, there was little evidence from meta-regressions to support this possibility. (DOCX) [file pone.0031660.s006.docx]

**Table S1: Meta-regression to investigate changes in the incidence of psychotic disorders in England over time**

| Outcome | Number of included rates (included citations / total identified citations)^1^ | Earliest mid-point^2^ | Latest mid-point^2^ | Time period (years) | Citation IDs  (primary citations in bold) | IRR  (95% CI; t-test p-value)^3^ |
| --- | --- | --- | --- | --- | --- | --- |
| All psychotic disorders | 8 (7/8)^4^ | 1979 | 2002 | 23 | 1,10,25,82,95,101, 109,113 | 1.01 (0.96, 1.06; p=0.63) |
| Non-affective psychoses | 9 (8/8)^5^ | 1984 | 2002 | 18 | 10,25,47, 93,95,109 | 1.00 (0.96, 1.53; p=0.97) |
| Schizophrenia | 15 (15/15) | 1965 | 2002 | 37 | 1,10,25,46,47,89,93, 56,70, 75,77,90,103,112,117 | 0.99 (0.95, 1.02; p=0.38) |
| Affective psychoses | 8 (7/7)^5^ | 1977 | 2002 | 25 | 1,10,25,70,76,90,95 | 0.98 (0.93, 1.03; p=0.39) |
| Bipolar disorder | 9 (7/9)^6^ | 1971 | 2002 | 31 | 25,76,93,49,68,95,98,100,121 | 1.00 (0.98, 1.03; p=0.64) |
| Depressive psychoses | 5(3/4)^7^ | 1977 | 2002 | 25 | 25,76,68,95 | 0.98 (0.93, 1.04; p=0.40) |
| Substance-induced psychoses | 4 (4/5) | 1979 | 2002 | 23 | 1,24,68,95,113 | 1.08 (0.95, 1.21; p=0.12) |

^1^Excluded citations were either secondary sources or did not provide a corresponding estimate of standard error with their rate

^2^Mid-point of case ascertainment period for the earliest/latest citation included in meta-regression

^3^IRR: incidence rate ratio; 95% CI: 95% confidence interval. IRR reports change in incidence per year

^4^One citation provided rate estimates from two separate studies [95: WHO, SIN], while one citation did not provide a rate with corresponding standard error [101]

^5^One citation provided rate estimates from two separate studies [95: WHO, SIN]

^6^Two primary citations provided rates each from two separate studies [95: WHO, SIN] [121: Salford Case Register study, Camberwell Case Register study]

^7^One citation provided rates from three separate studies [95: WHO, SIN, ÆSOP]
