# Supplementary material for: Incidence of Schizophrenia and Other Psychoses in England, 1950–2009: A Systematic Review and Meta-Analyses
Source: PLoS One. 2012 Mar 22;7(3):e31660. doi: 10.1371/journal.pone.0031660 (PMC3310436; doi:10.1371/journal.pone.0031660)
Supplement: Table S2 — Meta-regression to investigate changes in the incidence of psychotic disorders in England by urbanicity. We conducted random effects meta-regressions on available data on the overall crude incidence of various psychotic disorders to investigate whether there was any evidence to support a change in the incidence of disorders according to our measure of urbanicity. The table shows there was some support for an increasing crude incidence of non-affective psychotic disorders (including schizophrenia) with increased urbanicity, but not for other disorders including the affective psychosis and substance-induced psychotic disorders. (DOCX) [file pone.0031660.s007.docx]

| Outcome | Number of included rates (included citations / total identified citations)^2^ | Urbanicity rank (most urban/ least urban) | Citation IDs  (primary citations in bold) | IRR  (95% CI; t-test p-value)^3^ |
| --- | --- | --- | --- | --- |
| All psychotic disorders | 8 (7/8)^4^ | 1/36 | 1,10,25,82,95,101, 109,113 | 1.02 (0.99, 1.04; p=0.17) |
| Non-affective psychoses | 9 (8/8)^5^ | 1/36 | 10,25,47, 93,95,109 | 1.022 (1.017, 1.028; p<0.001) |
| Schizophrenia | 15 (15/15) | 1/36 | 1,10,25,46,47,89,93, 56,70, 75,77,90,103,112,117 | 1.03 (1.01, 1.03; p=0.01) |
| Affective psychoses | 8 (7/7)^5^ | 1/36 | 1,10,25,70,76,90,95 | 1.00 (0.95, 1.05; p=0.94) |
| Bipolar disorder | 9 (7/9)^6^ | 4/36 | 25,76,93,49,68,95,98,100,121 | 1.01 (0.99, 1.04; p=0.18) |
| Depressive psychoses | 5(3/4)^7^ | 21/36 | 25,76,68,95 | 1.02 (0.89, 1.16; p=0.73) |
| Substance-induced psychoses | 4 (4/5) | 21/36 | 1,24,68,95,113 | 0.97 (0.79, 1.18; p=0.55) |

**Table S2: Meta-regression to investigate changes in the incidence of psychotic disorders in England by urbanicity^1^**

^1^Each study setting (excluding national, regional and specialist settings such as prisons) was ranked according to the perceived level of urbanicity by four British content-area experts independently, including a geographer, sociologist and psychiatric epidemiologists. Ranks were averaged (mean) and re-ranked to provide a score from 1 (most urban) to 38 (least urban)

^2^Excluded citations were either secondary sources or did not provide a corresponding estimate of standard error with their rate

^3^IRR: incidence rate ratio; 95% CI: 95% confidence interval. IRR reports change in incidence per rank increase in urbanicity

^4^One citation provided rate estimates from two separate studies [95: WHO, SIN], while one citation did not provide a rate with corresponding standard error [101]

^5^One citation provided rate estimates from two separate studies [95: WHO, SIN]

^6^Two primary citations provided rates each from two separate studies [95: WHO, SIN] [121: Salford Case Register study, Camberwell Case Register study]

^7^One citation provided rates from three separate studies [95: WHO, SIN, ÆSOP]
