# Supplementary material for: Incidence of Schizophrenia and Other Psychoses in England, 1950–2009: A Systematic Review and Meta-Analyses
Source: PLoS One. 2012 Mar 22;7(3):e31660. doi: 10.1371/journal.pone.0031660 (PMC3310436; doi:10.1371/journal.pone.0031660)
Supplement: Table S3 — Meta-regression to investigate changes in the incidence of psychotic disorders in England by study quality. We conducted random effects meta-regressions on available data on the overall crude incidence of various psychotic disorders to investigate whether there was any evidence to support a change in the incidence of disorders according to study quality. Overall there was little evidence to support this, although we noted that higher quality studies tended to report a lower incidence of the depressive psychoses. (DOCX) [file pone.0031660.s008.docx]

| Outcome | Number of included rates (included citations / total identified citations)^2^ | Mean Study Quality (lowest/highest) | Citation IDs  (primary citations in bold) | IRR  (95% CI; t-test p-value)^3^ |
| --- | --- | --- | --- | --- |
| All psychotic disorders | 8 (7/8)^4^ | 4.8 (2/7) | 1,10,25,82,95,101, 109,113 | 1.07 (0.94, 1.22; p=0.25) |
| Non-affective psychoses | 9 (8/8)^5^ | 5.8 (2/7) | 10,25,47, 93,95,109 | 1.12 (0.97, 1.30; p=0.09) |
| Schizophrenia | 15 (15/15) | 4.8 (2/7) | 1,10,25,46,47,89,93, 56,70, 75,77,90,103,112,117 | 1.02 (0.88, 1.20; p=0.74) |
| Affective psychoses | 8 (7/7)^5^ | 4.5 (2/7) | 1,10,25,70,76,90,95 | 0.86 (0.71, 1.04; p=0.10) |
| Bipolar disorder | 9 (7/9)^6^ | 4.3 (2/7) | 25,76,93,49,68,95,98,100,121 | 1.05 (0.91, 1.21; p=0.42) |
| Depressive psychoses | 5(3/4)^7^ | 4.8 (2/6) | 25,76,68,95 | 0.81 (0.71, 0.93; p=0.02) |
| Substance-induced psychoses | 4 (4/5) | 6.0 (4/7) | 1,24,68,95,113 | 0.87 (0.35, 2.15; p=0.57) |

**Table S3: Meta-regression to investigate changes in the incidence of psychotic disorders in England by study quality^1^**

^1^Study quality was rated by consensus between two content-area experts [JBK & AE] and assessed on an 8-point scale (from zero to seven), with one point for evidence of each of the following: defined catchment area, accurate denominator source, population-based case-finding, standardised research diagnoses, attempt at blinding investigators to demographic variables, well-defined inclusion/exclusion criteria, leakage study.

^2^Excluded citations were either secondary sources or did not provide a corresponding estimate of standard error with their rate

^3^IRR: incidence rate ratio; 95% CI: 95% confidence interval. IRR reports change in incidence per point increase in reported study quality

^4^One citation provided rate estimates from two separate studies [95: WHO, SIN], while one citation did not provide a rate with corresponding standard error [101]

^5^One citation provided rate estimates from two separate studies [95: WHO, SIN]

^6^Two primary citations provided rates each from two separate studies [95: WHO, SIN] [121: Salford Case Register study, Camberwell Case Register study]

^7^One citation provided rates from three separate studies [95: WHO, SIN, ÆSOP]
