# Supplementary material for: Incidence of Schizophrenia and Other Psychoses in England, 1950–2009: A Systematic Review and Meta-Analyses
Source: PLoS One. 2012 Mar 22;7(3):e31660. doi: 10.1371/journal.pone.0031660 (PMC3310436; doi:10.1371/journal.pone.0031660)
Supplement: Box S1 — Description of study quality criterion. Each citation included in this review was rated by the authors (JBK, AE) according to seven study quality criterion we reasoned that, if reported, could be taken to indicate methodological rigor. Details of each criterion are provided here. (DOCX) [file pone.0031660.s009.docx]

**Box S1: Description of study quality criterion**

|  | Criterion | Description |
| --- | --- | --- |
| 1. | *Defined catchment* | Did the study have a well-defined catchment area to ensure that the numerator (case) and denominator populations came from the same source? Evidence of a defined catchment was needed to meet this criterion. |
| 2. | *Accurate denominator* | Did the study report how it estimated the denominator population from which the cases came? Was the source known to be accurate, reliable and valid? Evidence of an accurate denominator was needed to meet this criterion. |
| 3. | *Population-based case finding* | Did the study employ a population-based case finding approach? This includes identifying cases from community-based settings and service contact points, including primary, secondary and tertiary facilities. Studies which only considered hospital-based admissions, for example, would not meet this quality criterion as they would be likely to underestimate the true rate of disorder in the community. Evidence of a broad case-finding approach was needed to meet this criterion. |
| 4. | *Standardised research diagnoses* | Did the study use standardised research diagnoses to ensure that the cases met comparable diagnostic criteria for psychotic disorder? Studies reliant on clinician-led chart diagnoses may introduce bias into diagnoses given inter-clinician variation in making diagnoses. Applying standardised criteria, by using a method such as the Schedule for the Clinical Assessment in Neuropsychology [SCAN] or using OPCRIT­-generated diagnoses would reduce such problems. Evidence of attempts to standardise diagnoses was needed to meet this criterion. |
| 5. | *Blinding to demographic factors* | Did the study blind the person/panel who diagnosed cases to certain demographic characteristics? For example, ethnicity, where it has been argued that raised rates of psychosis in migrants and their offspring [11] may be due to misunderstanding of cultural differences in symptomatology or, worse, institutionalised racism. Blinding, or partially blinding diagnosticians to this information will reduce the likelihood of this explanation. Some evidence of blinding was needed to meet this criterion. |
| 6. | *Inclusion criteria* | Did the study use inclusion criteria to accurately define their study population (numerator and denominator)? Were these criteria sufficient? Standard criteria in epidemiological research include age limits, residency within catchment area at time of disorder, absence of an organic basis to the disorder and no previous episode of disorder (incidence studies only). Inclusion criteria needed to be present *and* of sufficient relevance/quality to meet this criterion. |
| 7. | *Leakage study* | Did the study conduct any kind of leakage study? A leakage study is an attempt by the investigators to identify any cases that may have been missed during original case identification. Leakage studies may be formal (systematic, regular consultation with mental health and other service providers in the region) or more informal (sampling a proportion of such services to estimate possible “leakage”). Evidence of any kind of leakage study was needed to meet this criterion. |
