# Supplementary material for: Incidence of Schizophrenia and Other Psychoses in England, 1950–2009: A Systematic Review and Meta-Analyses
Source: PLoS One. 2012 Mar 22;7(3):e31660. doi: 10.1371/journal.pone.0031660 (PMC3310436; doi:10.1371/journal.pone.0031660)
Supplement: Box S2 — Principle hypotheses to explain raised rates of psychotic disorder in migrant groups and their offspring. Here we summarize the main hypotheses that have been proposed to explain the excess incidence of psychotic disorders in migrants and their offspring. For each, we provide a description of the hypothesis, who originally proposed it and the evidence for and against. (DOCX) [file pone.0031660.s010.docx]

| **Box S2: Principle hypotheses to explain raised rates of psychotic disorder in migrant groups and their offspring** | **Hypothesis title** | **Hypothesis description** | **TYPE OF HYPOTHESIS^†^** | **Proposed by (YEAR)** | **Evidence for^‡^** | **Evidence against^‡^** | **Notes** |
| --- | --- | --- | --- | --- | --- | --- | --- |
| H1 | Predisposition to migrate | People at genetic disposition to psychosis were more likely to migrate | Reverse causality | Ødegaard [153] (1932) | Initial observations of Ødegaard [153] | Selten *et al*.’s natural experiment rejected hypothesis [160]  Raised rates in second- (& later-? i.e. 26) generation groups (i.e.10, 86,102,S1)  Migration highly complex task for people predisposed to psychosis [194] |  |
| H2 | High rates in sending country | Elevated rates in country of origin would explain higher rates in immigrants | Reverse causality | Cochrane & Bal [S2] (1987) | None | Incidence rates of schizophrenia in the Caribbean comparable to those in host UK & Dutch population [162,163,164,S3]. Hospitalised rates in Ireland higher than those for Irish migrants to UK [S2] | Few comparative studies have been conducted other than the UK vs. Caribbean studies noted. Irish comparative study [S2] only based on hospitalized rates. Other studies would be informative i.e. rates in Morocco vs. Moroccan migrants in the Netherlands |
| H3 | Socio-demographic differences | Age, sex, martial status & socioeconomic status [SES] differences between host & immigrant groups explain differences | Confounding | Cochrane & Bal [S2] (1987) | Young, male groups over-represented in initial migrant groups. Also known to be at increased risk of psychosis [9] | Control for age & sex [10,73,86,117,149,157,S1,S4,S5, latterly SES [26,155,156]. Marital status a consequence, not cause of psychosis [S2] |  |
| H4 | Misdiagnosis of psychotic symptoms | Psychiatrists in host country may misdiagnosis psychotic symptoms in migrant groups, unfamiliar with their socio-cultural norms, or tendency to over-diagnosis migrants with schizophrenia vs. other psychotic disorders | Bias | Cochrane & Bal [S2] (1987) | Early evidence of institutionalized racism in mental health services [165], particularly with regard to pathways to care [S6].  Psychotic symptoms may be more prevalent in Caribbean migrants [S7]  Poor inter-rater reliability between English & Jamaican psychiatrist [166] | Standardized diagnoses used in research, often quasi-blind to ethnicity of subject [1].  Raised rates of psychotic disorders not limited to schizophrenia [10, 73,120].  Inter-rater reliability was poor but not racially biased [166] | Rates of psychotic disorders in migrants persisted despite improved study designs & standardized diagnoses. Separate to the problem of institutionalized racism – see [150] for controversies surrounding this area. Cultural variation in symptom interpretation needs further research |
| H5 | Migratory & post-migratory factors | Several, but involving negative consequences of migration, acculturation & post-migratory living as relevant. Stress/vulnerability is posited as potential biological mechanism. | Confounding | Cochrane & Bal [S2] (1987),  Bhugra [S8,S9] (2000, 2004),  Jones & Fung [S10] (2005) | Ethnic density effect implicates social support as protective [54,94,171]  Higher rates of psychosis in BME groups which experience greater discrimination [168]  Neighbourhoods with more ethnic fragmentation have higher rates of psychosis [97]  Social adversity confounds relationship between psychosis & migrancy [S11]  Greater impact of social disadvantage in black Caribbean migrants than white British [182] | Other purportedly stress-induced disorders not raised for immigrants (i.e. depression) [S2,S12]  Immigrants experience similar levels of stress but variation in rates of psychosis is marked [S2]  Not conclusive evidence on discrimination [170] | Cochrane & Bal’s [S2] assertion that experience of migratory factors is similar across all immigrants is unlikely to now hold given likely genetic variation in stress vulnerability & differential experiences of migration along other socio-demographic & -cultural dimensions (i.e. family structure, social support, networks) |
|  | **Hypothesis title** | **Hypothesis description** | **TYPE OF HYPOTHESIS^†^** | **Proposed by (YEAR)** | **Evidence for^‡^** | **Evidence against^‡^** | **Notes** |
| H6 | Life course factors & neuro-development | Factors across the life course, including pre- & peri-natally, and through childhood have greater impact in migrants. Includes vitamin D hypothesis: a change in maternal vitamin D exposure after migration alters offspring neurodevelopment | Confounding | Eagles [S13] (1991),  McGrath [S14],  Jones & Fung [S10] (2005) | Separation from parents during childhood has greater impact in black Caribbean migrants than white British [183]  Prenatal hypovitaminosis D associated with schizophrenia risk in general [193], but… | No evidence that pre - & peri-natal problems have greater role in migrant than native groups [S12]  No current evidence directly linking migration, hypovitaminosis & psychosis | Evidence is mixed, depending on type of risk factor & period of life course. Further research required. |
| H7 | Substance abuse | Greater substance misuse in migrants accounts for higher rates | Confounding | Jones & Fung [S10] (2005) | None | Little evidence cannabis used more in black Caribbean than white patients [S15] or general population (i.e. [S16,S17,S18], or substance use more generally [S19]) | Putative link between cannabis & schizophrenia [146] combined with misconception that cannabis consumption was more prevalent in black Caribbean fuelled “hypothesis” |
| H8 | Psychological hypotheses | Interpretation of life events have greater impact on psychosis in migrant groups | Mediating factor | Jones & Fung [S10] (2005) | Tendency to attribute life events to an external locus may lead to onset of paranoid symptoms in some migrant groups. Evidence is weak [S20]. | No differences in number of life events experienced by UK white vs. black Caribbean migrants [S21] | Difficult to exclude this hypothesis & may mediate or have some overlap with other hypotheses (i.e. H5, H6, H10). |
| H9 | Genetic predisposition | Genetic factors explain higher rates in migrant groups | Genetic confounding | Jones & Fung [S10] (2005) | None | Morbid risk is similar for offspring of both black Caribbean migrants & white group in UK [S22,S23]. Larger morbid risk in second-generation migrants suggests environmental, not genetics pressures alone.  Rates of psychosis in Caribbean comparable to those in host UK population [162,163,164] | Genetic factors alone are unlikely to explain differences in rates between migrants & host population but genetic susceptibility in combination with environmental exposures (i.e. interaction – see Hypothesis 10 might be important) |
| H10 | Gene-environment interactions & epigenetic processes | People with underlying susceptibility genes for psychosis at increased risk if exposed to stressful environmental factors i.e. migration & other post-migratory factors. May be regulated epigenetically i.e. changes to gene expression following changes to environmental stimuli after migration | Interaction | Rutter [S24] (2002),  Broome *et al.* [S25] (2005),  Dealberto [S26] (2007) | Little explicit evidence either way. Ethnic density effect is proxy for interaction between individual phenotype (i.e. BME status) & exposure to environmental stressors (i.e. 54,94,171). No direct study of genes vs. environment in psychosis & migrants but studies are underway [S27] | Little explicit evidence either way. | Promising avenue for future research. More studies required. |

**Supplemental References for Box S2**

S1. Veling W, Selten JP, Veen N, Laan W, Blom JD, et al. (2006) Incidence of schizophrenia among ethnic minorities in the Netherlands: A four-year first-contact study. Schizophr Res 86: 189-193.

S2. Cochrane R, Bal SS (1987) Migration and schizophrenia: an examination of five hypotheses. Soc Psychiatr 22: 181-191.

S3. Hanoeman M, Selten J-P, Kahn RS (2002) Incidence of schizophrenia in Surinam. Schizophrenia Research 54: 219-221.

S4. Harrison G, Brewin J, Cantwell R, Dalkin T, Fox R, et al. (1996) The increased risk of psychosis in African-Caribbean migrants to the UK: a replication. Schizophrenia Research 18: 102.

S5. Selten JP, Veen N, Feller W, Blom JD, Schols D, et al. (2001) Incidence of psychotic disorders in immigrant groups to The Netherlands. British Journal of Psychiatry 178: 367-372.

S6. Morgan C, Mallett MR, Hutchinson G, Bagalkote H, Morgan K, et al. (2005) Pathways to care and ethnicity I. Sample characteristics and compulsory admission: Report from the ÆSOP study. Br J Psychiatry 186: 281-289.

S7. Johns LC, Nazroo JY, Bebbington P, Kuipers E (2002) Occurrence of hallucinatory experiences in a community sample and ethnic variations. Br J Psychiatry 180: 174-178.

S8. Bhugra D (2000) Migration and schizophrenia. Acta Psychiatrica Scandinavica 102: 68-73.

S9. Bhugra D (2004) Migration and mental health. Acta Psychiatr Scand 109: 243-258.

S10. Jones PB, Fung WLA (2005) Ethnicity and Mental Health: The Example of Schizophrenia in the African Caribbean Population in Europe. In: Rutter M, Tienda M, editors. Ethnicity and causal mechanisms. Cambridge: Cambridge University Press.

S11. Hjern A, Wicks S, Dalman C (2004) Social adversity contributes to high morbidity in psychoses in immigrants -a national cohort study of two generations of Swedish residents. Psychol Med 34: 1025-1033.

S12. Sharpley M, Hutchinson G, McKenzie K, Murray RM (2001) Understanding the excess of psychosis among the African-Caribbean population in England. Review of current hypotheses. Br J Psychiatry Suppl 40: s60-68.

S13. Eagles JM (1991) The relationship between schizophrenia and immigration: Are there alternatives to psychosical hypotheses? Br J Psychiatry 159.

S14. McGrath J (1999) Hypothesis: is low prenatal vitamin D a risk-modifying factor for schizophrenia? Schizophr Res 40: 173-177.

S15. McGuire PK, Jones P, Harvey I, Williams M, McGuffin P, et al. (1995) Morbid risk of schizophrenia for relatives of patients with cannabis-associated psychosis. Schizophrenia Research 15: 277-281.

S16. Coulthard M, Farrell M, Singleton N, Meltzer H (2002) *Tobacco, alcohol and drug use and mental health*. London: HMSO.

S17. Sandwijk JP, Cohen PD, Musterd S, Langemeijer MP (1995) *Licit and Illicit Drug Use in Amsterdam. Report of a Household Survey in 1994 on the Prevalence of Drug Use among the Population of 12 years and over.* Amsterdam: University of Amsterdam.

S18. Sharp C, Budd T (2003) Minority ethnic groups and crime: findings from the Offending Crime Survey, 2003. Home Office Online Report 33/05. London: Home Office.

S19. Veen N, Selten JP, Hoek HW, Feller W, van der Graaf Y, et al. (2002) Use of illicit substances in a psychosis incidence cohort: a comparison among different ethnic groups in the Netherlands. Acta Psychiatrica Scandinavica 105: 440-443.

S20. Sharpley MS, Peters ER (1999) Ethnicity, class and schizotypy. Social Psychiatry and Psychiatric Epidemiology 34: 507-512.

S21. Gilvarry CM, Walsh E, Samele C, Hutchinson G, Mallett R, et al. (1999) Life events, ethnicity and perceptions of discrimination in patients with severe mental illness. Soc Psychiatry Psychiatr Epidemiol 34: 600-608.

S22. Hutchinson G, Takei N, Fahy TA, Bhugra D, Gilvarry C, et al. (1996) Morbid risk of schizophrenia in first-degree relatives of white and African-Caribbean patients with psychosis. Br J Psychiatry 169: 776-780.

S23. Sugarman PA, Craufurd D (1994) Schizophrenia in the Afro-Caribbean Community. British Journal of Psychiatry 164: 474-480.

S24. Rutter M (2002) The Interplay of Nature, Nurture, and Developmental Influences: The Challenge Ahead for Mental Health. Arch Gen Psychiatry 59: 996-1000.

S25. Broome MR, Woolley JB, Tabraham P, Johns LC, Bramon E, et al. (2005) What causes the onset of psychosis? Schizophrenia Research 79: 23-34.

S26. Dealberto MJ (2007) Why are immigrants at increased risk for psychosis? Vitamin D insufficiency, epigenetic mechanisms, or both? Medical Hypotheses 68: 259-267.

S27. EU-GEI (2008) Schizophrenia aetiology: Do gene-environment interactions hold the key? Schizophrenia Research 102: 21-26.
